# Identification of potential regulatory mutations using multi-omics analysis and haplotyping of lung adenocarcinoma cell lines

**DOI:** 10.1038/s41598-018-23342-1

**Published:** 2018-03-21

**Authors:** Sarun Sereewattanawoot, Ayako Suzuki, Masahide Seki, Yoshitaka Sakamoto, Takashi Kohno, Sumio Sugano, Katsuya Tsuchihara, Yutaka Suzuki

**Affiliations:** 1 Department of Computational Biology and Medical Sciences, Graduate School of Frontier Sciences, the University of Tokyo, Chiba, Japan; 2 Division of Translational Genomics, Exploratory Oncology Research and Clinical Trial Center, National Cancer Center, Chiba, Japan; 3 Department of Bioinformatics and Systems Biology, Faculty of Science, the University of Tokyo, Tokyo, Japan; 4 Division of Genome Biology, National Cancer Center Research Institute, Tokyo, Japan

## Abstract

The functional relevancy of mutations occurring in the regulatory regions in cancers remains mostly elusive. Here, we identified and analyzed regulatory mutations having transcriptional consequences in lung adenocarcinoma-derived cell lines. We phased the mutations in the regulatory regions to the downstream heterozygous SNPs in the coding regions and examined whether the ChIP-Seq variant tags of the regulatory SNVs and the RNA-Seq variant tags of their target transcripts showed biased frequency between the mutant and reference alleles. We identified 137 potential regulatory mutations affecting the transcriptional regulation of 146 RefSeq transcripts with at least 84 SNVs that create and/or disrupt potential transcription factor binding sites. For example, in the regulatory region of *NFATC1* gene, a novel and active binding site for the ETS transcription factor family was created. Further examination revealed that 31 of these disruptions were presented in clinical lung adenocarcinoma samples and were associated with prognosis of patients.

## Introduction

Recent advances in the next generation sequencing technology have widened our view on cancer genomes. Substantial number of so-called driver genes have been identified, and how mutations (single nucleotide variations; SNVs) in those genes disrupt the gene functions and drive carcinogenesis processes has been elucidated in a large number of cancers. For example, The Cancer Genome Atlas (TCGA) has reported genomic mutations in 33 different types of cancers in their initial paper^1^. They have revealed comprehensive patterns of mutation features in key pathways of many cancer types, including lung adenocarcinoma, that could be used as anti-cancer drug targets^2^. In the International Cancer Genome Consortium (ICGC)^3^ and other large-scale research projects, SNVs within coding regions functionally changing the activities of tyrosine kinase and other signaling molecules have also been intensively examined for their possibility as cancer driver mutations.

During the rapid progression in cancer genome analyses, previous studies have mainly focused on the mutations in the protein-coding regions which would invoke alterations in amino acid sequences. On the other hand, SNVs in non-protein-coding regions have not been focused on until recently. Despite the general lack of sufficient annotations on the mutations in the non-coding regulatory regions such as promoter and enhancer elements, recent studies have reported that such “regulatory mutations” should be no less important than coding mutations^4,5^. For example, TERT promoter mutations have been identified in a large number of patients with melanoma and other types of cancers^6,7^. These mutations changed the binding elements of oncogenic transcription factors and consequently enhanced TERT expression. In the ICGC study of liver cancer, whole-genome sequencing analysis of a 300 patients cohort have characterized comprehensive patterns of non-coding and structural alterations that could influence gene expression in cancerous cells^8^. Indeed, there have been several pioneering studies that have attempted to comprehensively identify such regulatory mutations. For example, in colon cancer, integrative analysis of genome and transcriptome data has been conducted^9^. However, there have been relatively rare cases in which the mutations in the potential regulatory regions could be systematically associated with the aberrant expression of their target genes^10^.

The roles of regulatory mutations could at least be examined when attempting to understand the aberrations occurring at the transcriptional level in the cancer. Deeper insights into these transcriptional signatures of the cancers may lead to comprehensive elucidations of yet undiscovered mechanisms underlying carcinogenesis, metastasis or drug-resistance, which may be related to the prognosis of patients or the identification of novel targets for pharmacological interventions. In our previous study, we generated a multi-omics sequencing catalogue of lung cancer cell lines, including whole genome, epigenome (DNA methylation and eight histone modifications) and transcriptome sequencing data^11^. We detected a number of somatic SNVs in regions considered to have promoter or enhancer functions. We attempted to distinguish functionally relevant regulatory SNVs from other neutral passenger SNVs. However, in our previous study, it was difficult to directly analyze the potentially functional regulatory SNVs regarding their transcriptome consequences. The difficulty was mostly derived from the fact that short-read sequencers had provided limited information. For most of the cases, it was impossible to associate the SNVs in the regulatory regions to SNVs in the downstream transcribed regions on the same allele, because the distance between them was beyond the reach of short-read sequencers.

To complement the drawback inherent to current short read sequencing technologies, we employed the recently developed GemCode technology (10x Genomics). This enables phasing of the human genome by intensively utilizing molecular barcoding technology^12^. In this method, large DNA fragments are confined in oil droplets together with gel-embedded barcodes (GEMs). By hybridization extension, each unique barcode is added to the DNA fragments within the droplet. Barcoded large DNA fragments are, then, mixed, sheared and then subjected to sequencing using Illumina short read sequencers. Long-read sequences originating from the large DNA fragments within a single droplet can be computationally assembled depending on the unique barcode and, by comparing variants on the different barcoded fragments, a large area of the genome could be anchored by those variants (SNPs) and thus “phased.” The reads used for anchoring the fragments are called “linked reads” and play a key role in this “synthetic long read sequencing” method.

Earlier studies mostly relied on genome-wide associations or chromatin immunoprecipitations to identify regulatory SNVs^13,14^. In this study, we included phasing of regulatory SNVs (which we identified in our previous study using Illumina sequencing) with their transcribed regions via GemCode technology. We also examined whether those SNVs have altered regulatory functions by examining any bias in allele expressions of downstream transcripts by RNA-Seq. For this purpose, we conducted a single-allele resolution expression analysis using ChIP-Seq and RNA-Seq data (see below for more details). We also generated and utilized TSS-Seq data^15^ to precisely determine the transcriptional starting sites (TSSs) in the target cells, which were essential for defining the regulatory and transcribed regions, as well as considering novel regulatory regions. Here, we describe the analysis of the functional consequences of SNVs located in possible transcriptional regulatory regions with regards to allele expression of their regulated transcripts.

## Results

### 10x GemCode synthetic long read sequencing enables phasing of lung adenocarcinoma cell lines

**Figure 1:**
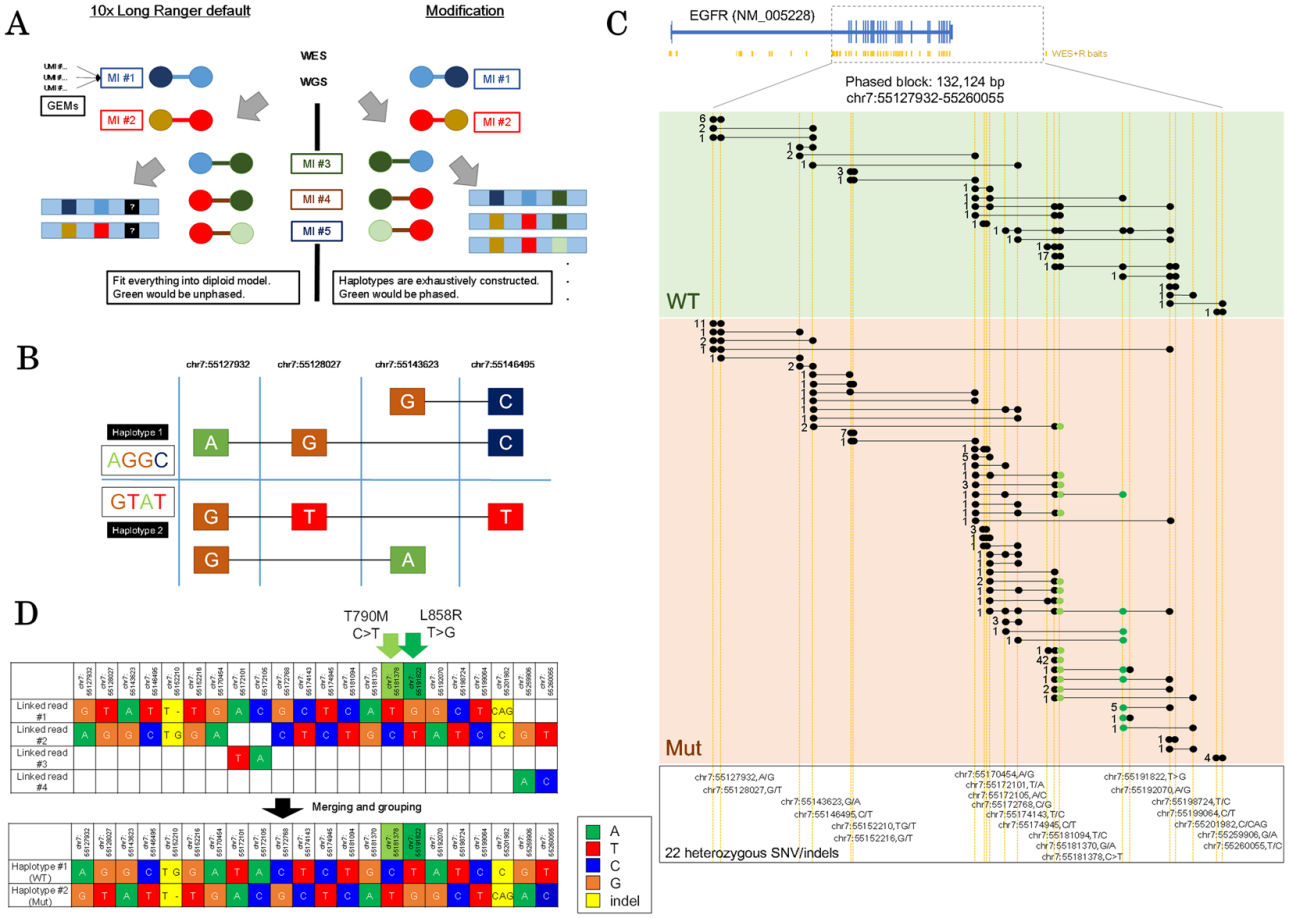
Phasing SNVs in the EGFR gene region. (**A**) The general premise of the phasing scheme. Haplotypes were exhaustively constructed to circumvent polyploidy/aneuploidy. (**B**) A simple example of successfully phased diploid haplotypes with connecting black lines indicating the original 10x GemCode linked read (MIs; molecular indexes). (**C**) Number of linked reads/molecular indexes supporting phased block partially covering the *EGFR* gene region in the H1975 cell line. The dots represent 22 heterozygous SNV/indels that could be completely phased in the both alleles. Somatic SNVs T790M and L858R are shown in light green and dark green, respectively. WT: wildtype allele; MUT: T790M/L858R mutant allele. (**D**) Details of each variant covered by phase block in (**C**). Upper panel is determined by direct connection via linked read/MIs. Lower panel is inferred by gaps and content of each redundant grouping of the linked read/MIs.

In our previous study, we constructed a multi-omics sequencing catalogue of a series of lung adenocarcinoma cell lines. We have investigated what types of genomic, epigenomic and transcriptional aberrations are present in a total of 23 cell lines; these aberrations span diverse driver mutation patterns in the protein-coding regions^11^. We also identified an average of 46,407 mutations in the regulatory regions identified by eight active or repressive histone markers (H3K4me1, H3K4me3, H3K9me3, H3K9/14ac, H3K27ac, H3K27me3, H3K36me3 and Pol II) for each cell line (Supplementary Table S1). To further characterize these potential regulatory SNVs, we first attempted to associate regulatory SNVs with their transcribed regions based on the “phasing.” For this purpose, we applied the synthetic long read sequencing technology (10x Genomics GemCode) to the lung adenocarcinoma cell lines (Fig. 1A and B). We limited the target SNVs for the phasing to those called from whole-genome sequencing^11^ (see Material and Methods for the re-mapping procedure to the UCSC hg38; see also Supplementary Table S1 for more details). An average of 4,038,252 SNVs were used per cell line. For the GemCode system, we employed target-capture sequencing for whole-exome and regulatory regions (113.7 Mb), which were designed to include exons, promoters, enhancers and differentially methylated DNA regions based on the ENCODE project^16^. An average of 45,679,789 paired-end reads (53x average depths for the bait regions) were obtained per cell line (Table 1). Using the collected data, 10.8% of the SNVs called from Illumina whole-genome sequencing were phased by default 10x Genomics GemCode Long Ranger pipeline and 5.2% of eligible heterozygous SNPs/SNVs were phased using our developed phasing schemes (Table 1 and Supplementary Table S2), due to our generally conservative phasing parameters (Supplementary Fig. S1; see discussion section for more details).

**Table 1:** Summary of the 10x GemCode sequencing data and phasing.

Categories		Average of 23 cell lines
Number of Reads		91,269,545
Percentage of Mapped Read		99.40%
PCR Duplication		5.00%
Bait Coverage		99.20%
Average Depths		53.3
Default Long Ranger Phasing Statistics (unused)	Longest Phase Block	826,778
	N50 Phase Block	98,130
	SNPs Phased	10.80%
Implemented Phasing Scheme Statistics	Longest Phase Block	1,197,601
	Average Phase Block Length	54,367
	Number of Phase Block	7,004
	Heterozygous SNPs Phased	5.18%

Default Long Ranger phasing statistics were from the Long Ranger results with every SNPs from Illumina whole genome sequencing as pre-called SNPs. Employed phasing scheme only used heterozygous SNPs for phasing (see Material and Methods for details).

For example, in a phased block of 132,124 bp (chr7:55127932-55260055) covering *EGFR* gene region in H1975 cell line (Fig. 1C and D), a total of 22 heterozygous SNVs including the two drug-sensitive/resistant mutations L858R and T790M were successfully phased. From total of 527 molecular indexes (MIs), 278 MIs spanned more than one SNV. Of these, both the wildtype and mutant alleles were covered by 139 MIs.

### Phasing enables association between regulatory mutations and their transcripts

**Figure 2:**
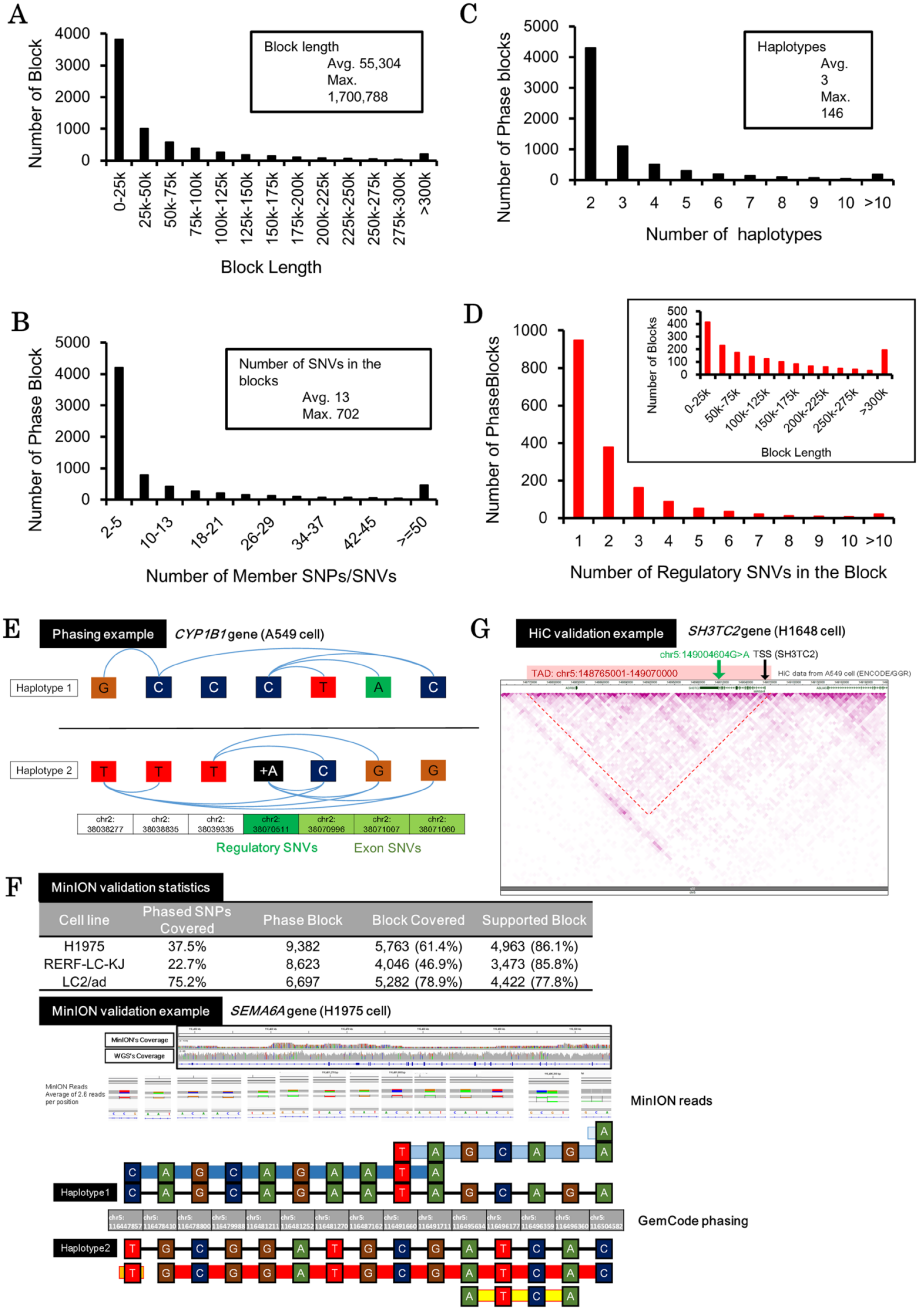
Phasing regulatory SNVs to the transcripts. (**A**) The distribution of phase blocks by length. The results were biased toward multiple, smaller blocks. (**B**) The distribution of phase blocks by number of member SNVs. The trends followed the length distribution in (**A**). (**C**) The distribution of phase blocks by reported haplotypes. (**D**) The distribution of phase blocks with regulatory SNVs by number of member SNVs. The distribution of the block length is shown in the inset. Blocks with regulatory SNVs showed no special characteristics or bias. (**E**) An example of the association between regulatory SNVs and its target transcript of the *CYP1B1* gene found in A549 cell line. Haplotypes were separated by black lines, and blue curved lines represent direct connection via linked read/MIs. (**F**) Validation of the phasing result by physical long reads sequenced from ONT MinION sequencing. Summary of the validation analysis is shown in the upper table for H1975, RERF-LC-KJ and LC2/ad cell lines. The lower panel shows one particular phase block in the H1975 cell line covering the *SEMA6A* gene region which was confirmed by both synthetic long read and physical long read phasing. Phasing from synthetic long reads is shown by thin black lines and that from physical long reads is shown by colored thick lines. (**G**) An example of TADs with regulatory mutations. A regulatory mutation within the same TAD of the TSS were visualized in A549 HiC data using the WashU EpiGenome Browser.

Based on the obtained phasing, we attempted to associate the SNVs in the regulatory regions with the SNPs/SNVs in the coding regions. From the GemCode analysis, we obtained an average of 7,004 phased blocks per cell line with mean length of 55 kb (maximum length: 1.7 Mb; Fig. 2A). Each phased block spanned an average of 13 SNVs (maximum: 702) and contained 3 haplotypes on average (Fig. 2B and C). An average of 3,884 (89,333 total) regulatory SNVs were associated with 1,742 (40,073 total) phased blocks, which were 130 kb in length on average. In these phased blocks with regulatory SNVs, an average of two regulatory SNVs were associated with a total of 33 SNVs (Fig. 2D). We further selected cases in which the regulatory SNVs were associated with the SNVs in the transcribed regions of the RefSeq transcripts. An average of 3,391 (78,006 total) regulatory SNVs in 1,389 (31,967 total) phased blocks per cell were associated with the SNVs in the transcribed regions of 3,018 RefSeq transcripts. For example, in the *CYP1B1* gene in A549 cell line, a regulatory insertion of C > CA (chr2:38070511) was phased to three SNPs in the transcripts (Fig. 2E). Namely, the C allele (reference allele) was phased to the T-A-C allele, while the CA allele (mutant allele) was phased to the C-G-G allele in the transcript region. Of note, we analyzed the phased blocks and found that these cells have an average of three haplotypes per cell line which was comparable to the public COSMIC database, which reported an average ploidy of 3.04 for 17 of 23 cell lines^17,18^ (Supplementary Fig. S2). While some cell line showed 1–1.5 ploidy difference with COSMIC, we considered these results, while not perfect, sufficiently accurate for the SNP association.

To more directly validate the phasing results, we conducted physical long read sequencing of the whole genome using MinION (Oxford Nanopore Technologies) in H1975, RERF-LC-KJ and LC2/ad cell lines. Due to its limited sequencing yield, we could map a total of 674,333 and 511,982 of mostly 2D reads from H1975 and RERF-LC-KJ, and 5,620,315 1D and 1D^2^ reads from LC2/ad to the UCSC hg38 human reference genome, which collectively covered 46%, 36% and 93% of the genome, respectively. Despite the limited overall coverage, these sequences covered or partially covered 5,763 (61%) phase blocks in H1975, 4,046 (47%) in RERF-LC-KJ and 5,282 (79%) in LC2/ad (Fig. 2F, see Supplementary Fig. S3 for sequencing depth and block coverage relation). Based on the combinations of SNPs/SNVs obtained, among the covered cases, we found that 4,962 blocks (86%), 3,473 blocks (86%) and 4,422 blocks (78%) were supported, respectively. With majority of the blocks supported consistently in all cell lines, we considered our phasing adequate for associating regulatory SNVs to their transcripts counterparts (see Supplementary Tables S3 and S4 for MinION sequencing runs).

We also validated the correct association between the enhancer and their target transcripts, using the previously published HiC data in A549 cell line in ENCODE dataset (see Method section for details). We found that in at least 21,177 (86%) of the cases, regulatory mutations and the TSS of their regulating transcripts were assigned to the same topologically associated domains (Fig. 2G; also see Supplementary Fig. S4 for further details).

### Whole transcriptomes sequencing revealed allelic transcriptional imbalance

**Figure 3:**
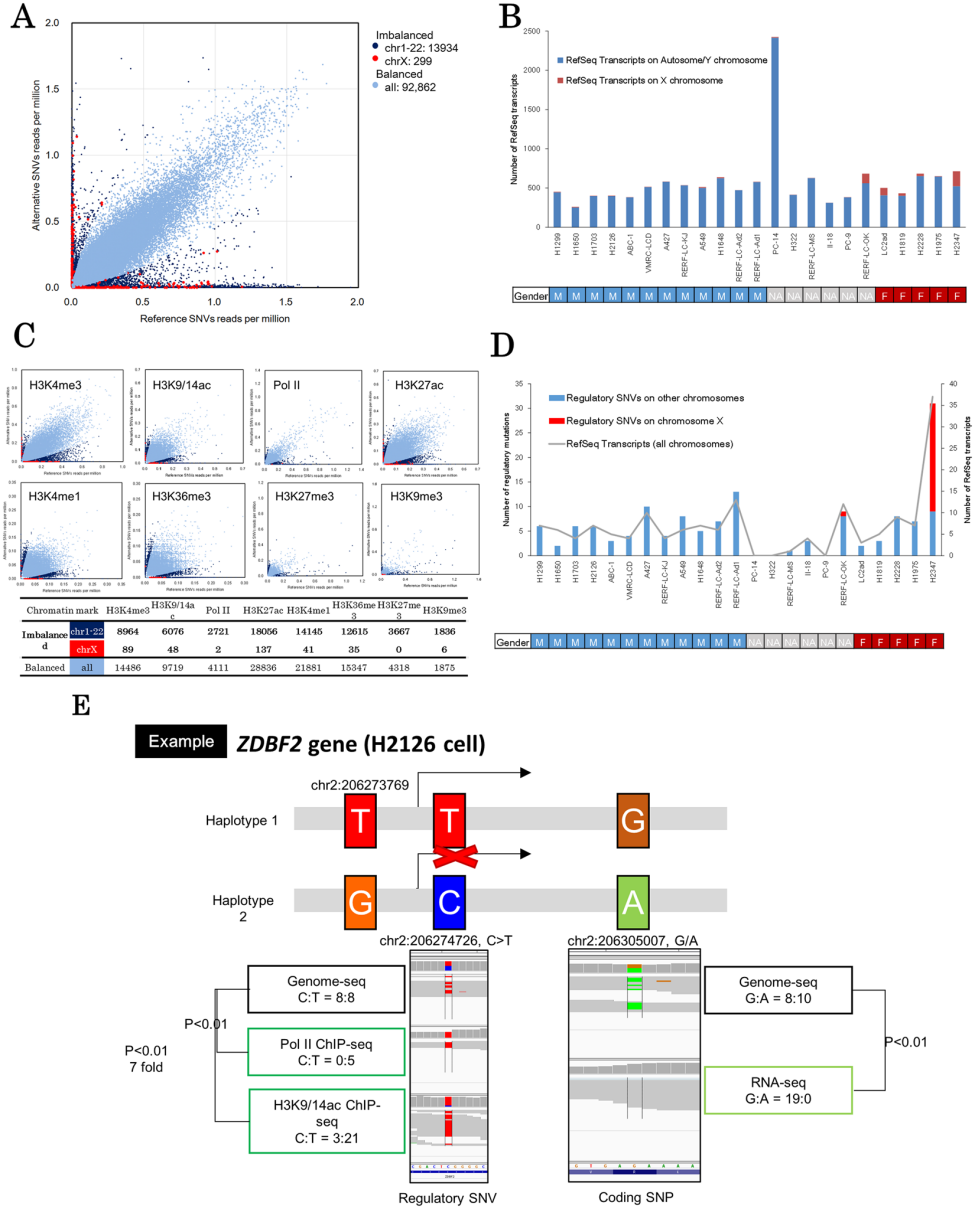
Allelic imbalance of transcriptional regulations. (**A**) Imbalance in SNP/SNV allelic expression (>5-fold changes, P < 0.01) detected in RNA sequencing. Red dots represent chromosome X imbalances. Dark blue dots represent other chromosome imbalances and light blue dots represent balanced expression. (**B**) Number of transcripts with imbalanced expression. Red bars represent chromosome X and blue bars represent other chromosomes. (**C**) Allelic ChIP imbalance of regulatory SNVs. Red dots represent chromosome X imbalances. Dark blue dots represent other chromosome imbalance and light blue dots represent balanced expression. The lower table shows the number of SNPs/SNVs in each category. (**D**) Histogram and line graph showing the number of imbalanced (both RNA and ChIP), phased RefSeq transcripts (gray line) and regulatory SNVs potentially influencing these transcripts (chromosome X in red, others in blue). (**E**) An example of regulatory mutations potentially causing biased transcript allele expression (*ZDBF2* in H2126). Pol-II and H3K9/14ac markers located in intron 1 showed bias in the ChIP-Seq and coding SNPs/SNVs in exon 5 showed expression bias in the transcriptomes.

We next examined which of these regulatory SNVs may affect the expression of their target transcripts. For this purpose, we utilized information on the allele-imbalanced expression of heterozygous variants in the transcribed regions. If the SNPs/SNVs-containing RNA-Seq tags were differentially represented between the alleles, it should be regarded as an indicator of those alleles being under mutually distinct regulations. When all the cell lines were taken together, we identified 107,155 (18,330 per cell line) SNVs in the coding regions of 29,251 RefSeq transcripts (1,271 per cell line) (Fig. 3A). Of these, 42,353 were considered heterozygous. Allele-biased expression was observed in 7,915 genes (596 per cell; also see Materials and Methods for the detailed selection procedure) via 5,419 SNPs (593 per cell) which accounted for 12% of all heterozygous SNPs (Supplementary Table S5).

To focus on cancer-associated regulatory SNVs, we removed cases in which allele-biased expression might be explained by other imprinting mechanisms, including sex-related or developmental lineage-specific repression of either of the alleles. It is true that the lineage-specific imprinting of these genes may also contribute to the unique patterns of genomic or transcriptional aberrations in lung adenocarcinoma compared with cancers of other lineages. However, for simplicity, we removed all of these cases in this study. For this purpose, we removed transcripts in which allele-biased expression was observed in more than 1/3 of the cell lines regardless of the diverse mutation patterns in the regulatory regions. A total of 124 transcripts in 76 genes were classified as imprinted transcripts (also see Supplementary Table S6 for the list and Supplementary Fig. S5 for GO analysis). The cases that were removed as possible “lineage or sex-imprinting” cases are also exemplified by the *BCLAF1* and *MAP2K3* genes in Supplementary Fig. S6. We further examined the cases of possible X chromosome inactivation/mono-allele in female and male cell lines. Cell lines of female origins showed a remarkably higher number of heterozygous variants on X chromosome (Fig. 3B and Supplementary Fig. S7) compared to those of male origins. We also noted that one cell line of unknown origin (RERF-LC-OK) is likely of female origin, based on the collected information. Interestingly, most of the cell lines, either male or female, did not exhibit complete imbalance/mono-allele patterns. Complex phenomenon(s) such as aneuploidy, copy number aberrations in male cell lines, or re-activation of the X chromosome in female cell lines may have taken place. Those aberrations may have occurred at the carcinogenesis stages of the cells or may have been induced during the long culture history of the cells.

We also looked for cases of known imprinting genes by referencing previously reported human imprinted genes^19^ and found a total of 67 transcripts in 17 genes. Thus, we concluded that the imprinting could be, at least partially, systemically observed by this approach.

### Multiple ChIP-Seq revealed an allelic imbalance in the regulatory regions

We took a similar approach to identify allele-biased usage of regulatory regions by using ChIP-Seq tags. We examined the biased representation of the ChIP-Seq tags in the enhancer or promoter regions defined by the “ChIP-Seq peaks” of eight chromatin markers (see Materials and Methods for details). Upon separately processing each of the chromatin markers and cell lines, we analyzed a total of 100,573 SNVs located in the promoter and enhancer regions of the 17,929 RefSeq transcripts (upper panels; Fig. 3C). We further selected SNVs that were associated with allele-biased transcriptions. We also removed potential imprinted genes detected earlier. As a result, a total of 1,794 SNVs in the regulatory regions (81 on average per cell line) were associated with 1,655 SNVs in the transcript regions in 730 RefSeq transcripts (38 on average per cell line). A breakdown of the number of the identified allele-biased regulated genes in each of the cell lines is shown in Supplementary Fig. S7.

### Identification of functionally relevant regulatory mutations

Finally, we integrated all the data from the phasing, allele-biased representations of RNA-Seq and ChIP-Seq. As a result, we identified a total of 146 RefSeq transcripts with 137 regulatory SNVs and 166 SNVs in the transcript regions (Fig. 3D). These genes were suggested to be subjected to aberrant transcriptional regulation due to the location of the mutations in the regulatory regions (Fig. 3D). For example, Fig. 3E illustrates the case of the *ZDBF2* gene in H2126 cell line. At the A/G heterozygous SNPs, only the transcripts from the “G” allele were observed while those from the “A” remained silent. Active chromosome markers (Pol II and H3K9/14ac) were found only on the “T” allele, while the “C” allele was silent. The “G” and “A” transcript alleles were phased to “T” and “C” alleles, respectively, which suggests that the regulatory mutation “C” is a functionally-relevant and transcriptionally silencing mutation. The complete list of all the “phased and allelic-biased” genes is shown in Supplementary Table S7.

We conducted GO overrepresentation analysis and found that genes related to many processes including “Regulation of gene expression, epigenetic” were significantly enriched (17.42 fold (5 genes); Benjamini p = 0.00285, Supplementary Table S8), suggesting that the identified regulatory mutations further expand their consequences by affecting the expression of their downstream targets.

**Figure 4:**
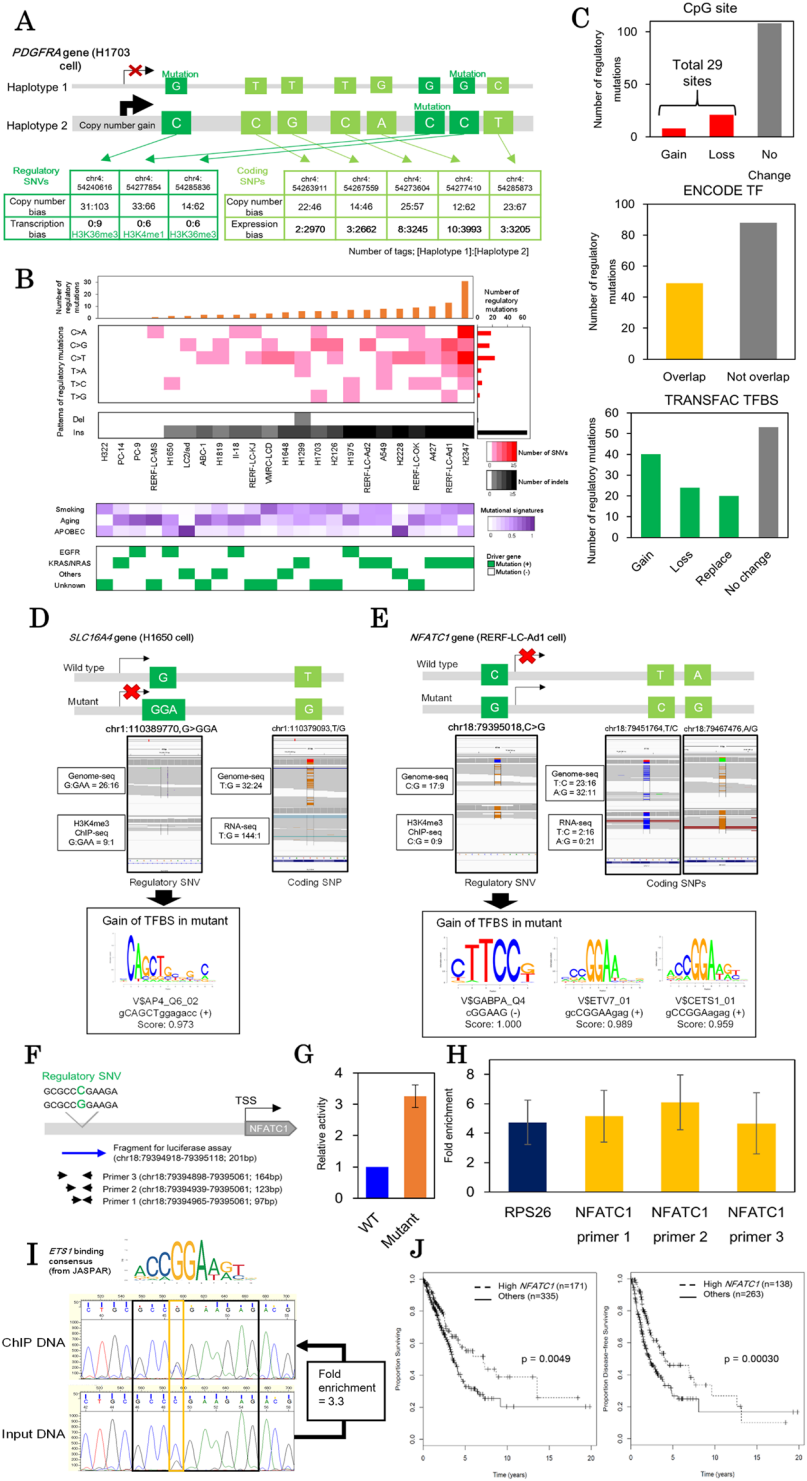
Regulatory mutations and aberrant transcription in lung cancer cell lines. (**A**) Allelic transcriptional and copy number bias of *PDGFRA* gene in H1703 cell line. Three regulatory mutations (green) and five coding SNPs (bright green) were phased. The number of sequencing tags in each haplotype were shown in small tables (upper: whole-genome sequencing; lower: ChIP-Seq or RNA-Seq). (**B**) Patterns of the regulatory mutations. The number of regulatory mutations were shown in the upper panel. Background mutational signatures are shown in the middle panel. Three background signatures (Smoking, aging and APOBEC signatures) were defined using somatic SNVs in coding regions. The lower panel shows known driver mutation statuses (*EGFR*, *KRAS* and *NRAS* mutations) for each cell line with color legends in the right margin. (**C**) Sequence contexts of regulatory mutations. Regulatory SNVs overlapping with the CpG sites, ENCODE ChIP-Seq peaks and TRANSFAC transcription factor binding sites were shown in the upper, middle and lower panels, respectively. (**D**,**E**) Examples of the regulatory SNVs in *SLC16A4* of H1650 cell line (**D**) and *NFATC1* of RERF-LC-Ad1 cell line (**E**). The regulatory mutation (green) and coding SNPs (bright green) were represented with the IGV (left). The changes of potential transcription factor binding sites are shown in the right panel. (**F**) The target region of the *NFATC1* upstream regulatory region. The blue arrow shows DNA fragment used for luciferase assays. Primers for ChIP-qPCR are shown as black arrows. Both the DNA fragment and primers covered the *NFATC1* regulatory SNV. (**G**) Luciferase assays for the *NFATC1* regulatory SNV. Relative activities of the *NFATC1* regulatory region were compared between wildtype (WT) and mutant sequences. Results were averaged from 3 biological replicates with 2 technical replicates each (n = 6) (**H**) ChIP-qPCR of *ETS1* in RERF-LC-Ad1 cell line (one primer for positive control *RPS26* and three primers for the *NFATC1* regulatory SNV as targets). Fold enrichments of ChIP DNA are shown in the graph. Results were averaged from 3 biological replicates with 2 technical replicates each (n = 6). See Fig. 4F for target regions of the *NFATC1* regulatory region and Supplementary Table S10 for primer sequences. (**I**) Direct Sanger sequencing of the *NFATC1* regulatory region in *ETS1* ChIP samples. Chromatogram of Sanger sequencing were shown in left (upper: ChIP DNA, lower: input DNA). The consensus sequence of *ETS1* binding sites is shown over the chromatogram. Fold enrichment of the mutant allele (“G”) compared with wildtype allele (“C”) is shown in the margin. (**J**) Kaplan-Meier analysis of cases in TCGA-LUAD data divided into two groups depending on the expression level of *NFATC1*. Overall survival and disease-free survival were shown in the left and right panels, respectively.

We also noticed that two cancer driver genes (*BCOR* and *PDGFRA*) were included among the identified genes^20^. The *BCOR* gene is located in the X chromosome and thus might be subjected to sex-related allelic imbalance. Intriguing observation was made in *PDGFRA* gene (Fig. 4A**;** see Supplementary Fig. S8 for haplotyping). It is known that some cases of lung cancers harbor mutational amplifications of this gene^21^. Particularly, H1703 cell line has allelic imbalanced copy number gains in *PDGFRA* and the amplified allele was transcriptionally activated via aberrant regulation. The data suggested that in this case the effect of regulatory mutation seems to be greater than that of copy number aberrations. It is possible that the combination of this alteration in the transcriptional regulations and the copy number gain could collectively contribute to carcinogenesis within its originating patient or the establishment of the cell line.

Moreover, we investigated the possibilities of disruptions of lncRNA and transcribed enhancer regions. We cross referenced the identified regulatory mutations with the FANTOM CAT v3 robust release^22^. We found that 31 from the 137 regulatory SNVs presented in regions associated with lncRNA (Supplementary Table S9A). Additionally, 5 of the 137 regulatory SNVs overlapped with the FANTOM5’s permissive enhancer region^23^ (Supplementary Table S9B).

### Cis-regulatory SNVs causing aberrant transcriptional regulations

We further characterized the 137 identified regulatory mutations. As shown in Fig. 4B, we found the regulatory SNVs showed an enriched pattern in C > T (G > A) and C > A (G > T) mutation signature. The C > T (G > A) substitution is a generally observed mutation pattern that is associated with 5-methyl cytosine deamination. In H2347 cell line, 11 C > A (G > T) substitutions were harbored (50% of the 22 regulatory substitutions in this cell line) and this signature is also a mutational pattern associated with smoking. These mutations were somewhat similar to those found in other genomic regions of these cell lines (general mutational signatures of each cell line were defined using coding SNVs)^24^. Remarkably, we found that one half of the identified regulatory mutations are insertions, which suggests that they have significantly distinct impact on the promoter and enhancer sequences. No significant correlations between the patterns of the regulatory mutations and driver genes were found.

We next examined whether the regulatory mutations affect that sequence contexts of specific regulatory motifs. We found that 29 regulatory mutations were located in CpG sites, which may affect DNA methylation status in the regulatory regions (upper panel, Fig. 4C).

In several cases, the regulatory mutations were also located in transcription factor binding sites. We examined the ChIP-Seq data of transcription factors (TFs) in A549 cell line using the ENCODE dataset^16^ and found that 49 of the identified regulatory mutations overlapped with the ChIP-Seq peaks (middle panel, Fig. 4C; also see Supplementary Table S7). For example, A427 cell line had an SNV in the promoter region (chr17:5191978 C > G) of the *ZNF594* gene and the mutant allele was transcriptionally silent (Supplementary Fig. S9). This regulatory SNV overlapped with several TF ChIP-Seq peaks including *POLR2A*, *TAF1* and *MYC*, according to the ENCODE dataset. This SNV is also located at 110 bp upstream of the transcription start site (chr17:5191868) as defined by TSS-Seq, suggested that the core promoter activity was silenced.

For more comprehensive analyses, we examined transcription factor binding consensus sequences using the TRANSFAC database, looking for possible disruption or acquisition of new motifs in the ±10 bp region of the identified regulatory SNVs^25^ and found 84 such regulatory SNVs (lower panel, Fig. 4C; also see Supplementary Table S7). For example, H1650 cell line harbored a promoter mutation (chr1:110389770, G > GAA) and this mutation created a binding motif for the AP-4 transcriptional factor (Fig. 4D). The mutant allele was transcriptionally silenced; thus this newly acquired TF binding might work as a transcriptional repressor. In another example, in RERF-LC-Ad1 cell line, a regulatory SNV (chr18:79395018 C > G) was found in the region of *NFATC1* gene. In this cell line, this gene showed almost complete allele-specific transcription from the mutant allele (Fig. 4E). The location of this SNV overlapped with the binding location of *RNF2* based on the A549 cell line analysis of the ENCODE data (left, Supplementary Fig. S9C). The *RNF2* gene encodes one of the polycomb group proteins and has a role in transcriptional repression, which suggests that *NFATC1* should be transcriptionally repressed via the recruitment of *RNF2* in A549 cell line (or other cell lines with wildtype *NFATC1* alleles). On the other hand, in this cell line, the regulatory mutation generated a novel binding site of the ETS family of transcription activators (CGGAAG, Fig. 4E).

To biologically validate this novel transcription factor binding site, we performed the luciferase assay of mutant and wildtype alleles and detected 3 fold increases in enhancer activity of the mutant allele compared to the wildtype allele (Fig. 4F and G). To solidify this result, we also conducted ChIP-qPCR for *ETS1*, a major transcription factor in the ETS family, targeting the region ±100 bps around the regulatory SNV. We detected 4–6 folds enrichments of the *ETS1* binding in the location of the regulatory SNV (Fig. 4F and H). Sanger sequencing of qPCR products also showed bias towards the mutant allele (Fig. 4I). We believe that these results ensured our notion regarding the creation of the ETS family binding site and supported the validity of our approach.

The *NFATC1* gene itself is also a transcription factor that is associated with immune responses. Even though expression level of the *NFATC1* gene in RERF-LC-Ad1 cell line is not particularly high (right, Supplementary Fig. S9C), previous studies reported that *NFATC1* promoted migration of the tumor cells^26^ and aberrant activations also correlated with tumor migration and invasion^27^. High expression of this gene also lead to poor prognosis in both patient and mice model^28^. However, when compared with overall survival rates of patients registered in TCGA database (506 case with overall survival data; 401 cases with disease-free survival data), good prognosis was associated with the high expression levels of the *NFATC1* (Fig. 4J). Future analyses of the mutation identified in this study should contribute to better understandings on the roles of the *NFATC1* in carcinogenesis.

Similarly, we found that expression levels of 31 genes in which regulatory mutations were identified in this study were associated with a better or worse prognosis (p < 0.05 with log-rank test; Supplementary Fig. S10). We believe these genes should be subjected to further in-depth study in order to elucidate the clinical relevance of the aberrant expression of these genes and their underlying molecular mechanisms.

## Discussion

In this study, using a series of lung adenocarcinoma cell lines, we identified a total of 137 SNVs or indels that are likely to have transcriptional consequences in the potential regulatory regions of 146 RefSeq transcripts. Among these, 104 SNVs were further characterized to change the binding consensus of transcription factor binding sites as defined by TRANSFAC or overlap with previous ENCODE ChIP-Seq analyses of transcription factors and chromatin remodeling factor binding. Particularly, we identified several regulatory aberrations that may also occur in clinical samples using TCGA datasets and a number of these aberrations also associated with prognosis of patients. We believe this is the first comprehensive attempt to systematically identify and characterize the SNVs having regulatory roles, and by using cell lines as starting materials, we were able to obtain large amount of multi-omics and long read data with relatively ease.

Despite the general success, we also noticed that the collected dataset is still far from complete. First, the population of the successfully characterized SNVs was limited to 137 out of a total of 1,794 SNVs (7.6%). These drops were mostly derived from the lack of solid information on phasing. In this study, to expedite the analysis, we utilized a hybridization capture method with bait covering only exon and regulome regions. However, recent analyses gradually revealed that many of the enhancer elements are tissue-type or occasionally cell-type specific^29^, thus, it was difficult to cover all the regulatory regions using singly designed capture panels. Additionally, we found that the GemCode Long Ranger software is not optimized for phasing non-diploid genomes as described in their original publication^12^. To circumvent the potential multi ploidy-related problem, we employed the re-assemble method by directly inspecting the phasing information using Molecular Indexes (MIs; see Materials and Methods for details) in 10x Long Ranger results. We achieved an average of 13 SNVs phased in 55 kb length block and 33 SNVs and 130 kb in the blocks of interest (i.e. regulatory SNVs), which we believe should be adequate for further analysis. Also, we observed an average of three haplotypes per cell line, which is comparable to reports in the COSMIC database^18^ (Supplementary Fig. S2). However, at individual cell line level, some discrepancies were observed. The discrepancy may have been derived from both over- and under-estimations of the haplotypes. Overestimation could arise from the conservative merging of the “local” SNPs, unless sufficient evidence supported the “merging” of the possible haplotypes, we left the haplotypes “un-merged.” Underestimation may have happened when only a part of the haplotypes were represented by the detected SNPs. At the same time, we employed generally strict parameters to avoid miss-identifications and strictly utilized only SNPs to SNPs associations which were not subjected to the problems of haplotype assembly. Indeed, we took a conservative approach, since we were concerned that the genes which may have the largest biological significance would reside in the regions of aneuploidy or regions with complicated copy number aberrations. To more thoroughly address this issue, carefully designed novel algorithms that incorporate other modes of detection should be developed.

Second, the currently available information of the transcription binding sites is still limited. We could examine the 50 ENCODE ChIP-Seq datasets that encompass a total of 41 types of transcription factors. Still, 24% of the identified potential regulatory SNVs remained elusive regarding their responsive trans-factors even though putative involvement of particular transcription factors is inferred from the binding consensus searches.

We consider this work a proof of concept study that could serve as a gateway towards better understanding of transcriptional regulatory aberrations in cancers. Indeed, in-depth understanding of the biological relevance of the aberrant transcriptional regulation, namely, how a large number of non-coding mutations that are being detected in cancer genomes collectively contribute to the etiology of cancers, may also serve as a potential approach for developing better therapeutic methods based on these completely novel concepts. Further technical development will allow us to directly collect transcriptome and epigenome data from clinical samples and long read technologies are also making rapid progress, which would enable more comprehensive studies. By first providing a link between the untracked regulatory alterations and the aberrant transcriptomic consequences in cancers, this study has provided a stepping stone towards that goal.

## Material and Methods

### Cell lines

All human lung cancer cell lines and small airway epithelial cells (SAEC) were cultured and harvested as previously described^11^.

### Synthetic long reads library preparation by 10x GemCode

From the 23 cell lines, high molecular weight DNA was extracted and quantified using Qiagen MagAttract HMW kit according to manufacturer’s recommendations (10x Genomics, Qiagen #67653). For each cell line, 1 × 10^6^ cells were suspended in 200 μl of PBS buffer, 20 μl of Proteinase K. Mixture, 4 μl of RNAase A and 150 μl of buffer AL.The samples were then incubate at 25◦C for 30 minutes. Approximately 15 μl of Qiagen MagAtrract suspension G were added to each sample along with 280 μl of buffer MB. The samples were mixed and centrifuged at 1400 rpm at 15–25 °C for 3 minutes. To wash the beads, the samples were placed on the magnetic rack for 1 minute and the clear supernatant was discarded. The beads were removed from the magnetic rack, suspended in 700 μl of Buffer MW1, mixed and centrifuged at 1400 rpm at 15–25 °C for 1 minute.The samples were placed back onto magnetic rack and the wash procedure was repeated once. After washing with Buffer MW1, the samples were then washed twice with 700 μl of Buffer PE. The beads with Buffer PE were placed on the magnetic rack for 1 minute. The supernatants were removed on the magnetic rack, and 700 μl of nuclease-free water was added and incubated for 60 seconds. After centrifugation, supernatants were discarded, and the processes were repeated once. After the beads were washed with Buffer MW1, PE and nuclease-free water twice, the beads were removed from the magnetic rack and 150 μl of Buffer AE was added to the bead pellets. The samples were mixed and centrifuged at 1400 rpm at 15–25 °C for 3 minutes. The samples were put back on the magnetic rack and held for 1 minute. The supernatants were transferred and stored at 4 °C for DNA quantification by Qubit dsDNA HS Assay kit (Thermo Fisher Scientific) with the target concentration set at 10–20 ng/μl.

For GemCode library preparation, partitioning was performed using GemCode Gel-Beads and Chip (10x Genomics). Indexing and library preparation was performed using a GemCode library preparation kit (10x Genomics) according to the manufacturer’s instructions. In brief, quantified high molecular weight DNA was further diluted with nuclease-free water to a concentration of 1 ng/μl, and 1.2 μl were used. The sample mix was prepared by adding 1.2 μl of diluted genomic DNA to the master mix, which consisted of nuclease-free water, GemCode Reagent Mix, Primer Release Mix and GemCode Polymerase supplied in the GemCode Reagents Kits. The Sample Mix, Gel beads and partitioning Oil were applied onto a GemCode Chip. The GemCode Chip was loaded in to the GemCode instrument.

Gel beads in emulsions (GEMs) were retrieved from the instrument according to the manufacturer’s recommendations and transferred to a 96-well plate for a designated thermal cycling amplification. For the post cycling recovery, 1 μl of Additive 1 and 125 μl of Recovery Agent were added and mixed with each GEM sample according to the manufacturer’s instructions. The aqueous solutions were transferred and the Recovery Agent and Partitioning Oil were removed. A mixture of the Recovery Agent and Partitioning Oil at the bottom was first removed by 135 μl of pipetting. The remainder was removed with DynaBeads MyOne SILANE beads and a 0.6x SPRI solution on the GemCode magnetic rack. The beads were washed with Elution Buffer I (Elution Buffer, 10% Tween-20, Additive 2), twice with SPRI reagent and once with Elution Buffer II (Elution Buffer, Additive 2).

The barcoded samples were subjected to library construction by shearing using a Covaris system. The fragmentation was performed with a target peak of 250 bp for whole exome and regulome sequencing. End repair and A-tailing were performed by thermal cycling the fragmented DNA with the End Repair and A-Tailing Buffer and Enzyme Mix supplied in the GemCode library preparation kits (10x Genomics). Products from the end repair and A-tailing were ligated by thermal cycling with Adaptor Mix and DNA Ligase. Post ligation cleanups were performed using 0.8x SPRI solution on the GemCode magnetic rack. Sample indexing PCR with the P5 primer were conducted. Post PCR cleanups were performed using 1.0x SPRI cleanup on the GemCode magnetic rack.

Target enrichment was performed using Agilent SureSelectXT protocol with SureSelect V5 plus regulome bait according to the manufacturer’s instructions (Agilent and 10x Genomics). The obtained products were sequenced on an Illumina Hiseq. 2500. The FASTQ files were processed using 10x Genomics LongRanger (version 1.3) pipeline on default setting together with the pre-called SNPs.

### Multi-omics datasets for each cell line

For each cell line the FASTQ files for whole-genome sequencing; chromatin immunoprecipitation sequencing (ChIP-Seq) for H3K9me, H3K9/14ac, H3K4me3, H3K4me1, H3K36me3, H3K27me3, H3K27ac, RNA Polymerase II and input DNA; whole transcriptome sequencing (RNA-Seq) and transcriptional starts site sequencing (TSS-Seq) were retrieved from a previous publication^11^. Annotations for the coding regions were obtained from the KERO database for UCSC hg38 human genome reference (http://kero.hgc.jp/)^15,30^.

### SNVs from whole genome sequence data

The FASTQ files for whole genome sequencing in each cell line were re-mapped to the UCSC hg38 human genome reference^31^ using bwa^32^ (version 7.15) with an aln algorithm on the default setting. PCR-duplicates were then removed using SAMtools^33^ (version 1.18). SNVs were called by GATK^34^ (version 3.3) HaplotypeCaller with the default parameters. The SNVs called by GATK with more than 5 supporting tags and a variant frequency greater than 5% were selected. The variant frequencies were calculated by SAMtools (v1.18) mpileup command with default setting.

### TSS-Seq

Total RNA from 27 cells lines (26 lung cancer cell lines and SAECs) was extracted using an RNeasy Maxi kit (QIAGEN). TSS-Seq libraries were created as previously described^35,36^ and sequenced using HiSeq. 2500 (Illumina) according to the manufacturer’s protocol. Sequences from the TSS-Seq data were mapped in human reference genome (UCSC hg19) using ELAND (Illumina). An average of 32,315,446 TSS-Seq tags were mapped and 84% of the mapped sequences were located on the proximal regions of the annotated start sites (up to 50 kb upstream from start sites and the first exon of the RefSeq transcripts). We constructed 12,248 TSS clusters (TSCs) in each cell by clustering the TSS-Seq tags to 500-bp windows and found that 69% of the TSCs were assigned as RefSeq TSCs. RefSeq TSCs from the 27 cells lines were merged considering their mutual overlaps of 500-bp bins. We finally obtained 24,008 TSCs which were used to determine a representative TSS for each gene.

### Regulatory regions defined by ChIP-Seq

ChIP-Seq data for 7 histone modifications (H3K9me, H3K9/14ac, H3K4me3, H3K4me1, H3K36me3, H3K27me3 and H3K27ac) and RNA Polymerase II were processed. The FASTQ files were re-mapped to the UCSC hg38 human genome reference using bwa (version 7.15) aln algorithm with default setting. PCR-duplicates were then removed using SAMtools (version 1.18). Each dataset peak was calculated by MACS2^37^ boardpeak calling with default parameters against input DNA as the background control. Peaks that were within 150 kb of transcriptional start site according to TSS-Seq data were treated as regulatory regions. If there were multiple transcriptional start sites, the closest transcriptional start site was selected for the peak. SNVs that were within the peaks were then defined as regulatory SNVs. The number of regulatory SNVs were collectively counted, if any SNVs were associated with multiple peaks, these SNVs would be counted multiple times and treated as separate SNVs.

### RNA sequencing

FASTQ files for RNA-Seq were re-mapped to the UCSC hg38 human genome reference using GSNAP with the default parameters^38^. Splice sites and introns were extracted from the DBKERO database.

### Background Germline Variants Filtering

SNVs called by GATK in the whole genome sequencings that were within the regulatory regions were treated as candidates for regulatory SNVs, which were filtered using NCBI’s dbSNP v142 (note that background germline SNPs were not available)^39^.

### Non-diploid phasing analysis

The method developed for this study was based on an exhaustive approach in merging multiple overlapping molecular indexes (MIs) together to create each haplotype. First, relationships between heterozygous SNVs called in conventional whole genome sequencing and MIs from the 10x GemCode were established by constructing an index of SNVs with respect to the MIs (i.e., which grouping of SNVs were supported by each MI) and vice versa (how many MIs were supporting each SNV) by cross-referencing lists of SNVs called by GATK from whole genome sequencings to the 10x GemCode Long Ranger (version 1.3) bam file. Using an exhaustive approach, we merged compatible MIs together to form a “preliminary haplotype”; MIs were deemed compatible if at least one SNV position overlapped and the nucleotide base matched. MIs with different bases were not merged into the same “preliminary haplotype” but would be designated another “preliminary haplotype” in the same phase block; this process would be done exhaustively until every MI was considered and each MI could be a member of more than one haplotype. Only reads with score > 20 and SNVs with score > 20 (if applicable) were considered.

Due to randomness in the distribution of barcoding coverage, it was possible that two adjacent SNVs in the same phase block would not share a preliminary haplotype even after all possible merging. Practically, this would result in phase block with multiple short and isolated preliminary haplotypes, which would not be very useful. To address this, we performed a second merging of the preliminary haplotype in the same phase block to create a final “haplotype” for that phase block via a greedy approach. First, by comparing each “preliminary haplotype” to the associated phase block overall coverage, each preliminary haplotype’s missing genomic positions (if any) were determined; then, for preliminary haplotypes with missing positions (starting from those with the fewest) we searched for the most similar haplotype that could fill the gaps. Similarity was determined by the number of compatible SNVs subtracted by the number of un-compatible SNVs, 0 was set for pairs without SNVs occupying the same genomic positions. The process would be repeated until the final haplotype was compete (no missing positions) and no more preliminary haplotypes remained. The results (final haplotypes) were then used in further analyses (Supplementary Fig. S1 for graphic representation).

### Analysis of HiC data

HiC data of A549 cell line (ENCSR662QKG) was obtained from the ENCODE/GGR dataset (https://www.encodeproject.org/)^16^. The files of chromatin interactions (.hic; biological replicate 1; ENCFF121YPY) and topologically associated domains (.bedpe; biological replicate 1–4; ENCFF513HKS) were downloaded and used in the analysis. The chromatin interactions was visualized by the WashU EpiGenome Browser^40^.

### Analysis of allelic expression imbalance in RNA-Seq and ChIP-Seq

Each allele was considered valid when the SNVs were called in the whole genome sequencing and total variant tag frequency assessed by SAMtools (version 1.18) mpileup was greater than 4. Bias towards each variant expression was calculated from each tag, normalized by total reads per million in each cell line and then log10 transformed with +1 adjustment for plotting. Allele expression was considered biased when one variant showed more than a 5-fold expression ratio compared with tags in the whole genome sequencing. Indels called from the whole genome sequencings were also manually re-checked for mapping error in the FASTQ file of ChIP-Seq datasets.

### Validation by physical long-read sequencing of MinION

HMW DNAs from cell lines were extracted from the cell pellets as described above. Sequencing libraries of long-read WGS for MinION were prepared according to the manufacturer’s instructions. gDNA was selected for long reads using SQK-LSK208 for 2D, SQK-LSK108 for 1D and SQK-LSK308 for 1D^2^ (Oxford Nanopore Technologies). In summary, 5 μg of HMW gDNAs were quantified using Qubit. DNA repair was performed using NEBNext FFPE Repair Mix (M6630, NEB). End-prep was performed using a NEBNext End repair/dA-tailing Module (E7546, NEB) and end-prepped DNAs were purified using Agencourt AMPure XP beads (Beckman Coulter). Adapter ligation and tether attachment were conducted using the NEBNext Blunt/TA Ligase Master Mix (M0367S, NEB) and Ligation Sequencing Kit SQK-LSK208 for 2D, SQK-LSK108 for 1D and SQK-LSK308 for 1D^2^. Libraries were then purified using MyOne C1 beads (65001, Thermo Fisher Scientific) and sequenced for 48 hours by MinION Mk 1B with the SpotON Flow Cell (FLO-MIN106, R9.4 version for 2D; FLO-MIN107, R9.5 version for 1D and 1D^2^, Oxford Nanopore Technologies).

FASTQ files were generated from the FAST5 files using poretools^41^. Sequences were mapped using bwa-mem with ont2d settings for 2D reads (H1975, RERF-LC-KJ) and default settings for 1D and 1D^2^ reads (LC2/ad).

MinION reads were considered if mapping quality scores were over 10. Every Phased SNPs were then check for coverage by MinION. For reads that spanned more than 1 SNPs position, combinations of SNPs configurations in every phase blocks’ haplotypes were checked against MinION reads. Due to presences of sequencing error in MinION reads (90% identity in 2D and 80% in 1D + 1D^2^), we considered any block that at least more than twice supportive reads compared to unsupportive reads to be supported by MinION sequencing. Additionally for 1D + 1D^2^ runs, we only considered nucleotides with base call quality over 15.

### Functional annotations for regulatory mutations

Background mutational signatures of the cell lines (middle panel, Fig. 4B) were examined using coding mutations. Three stable signatures (stability > 0.9) were extracted using the WTSI mutational signature framework^42^ based on non-negative matrix factorization. These signatures were annotated by comparing known mutational signatures in cancers^24^.

Gene ontology overrepresentation analysis was conducted using PANTHER classification version 11.1 with PANTHER GO Slim (version 11.1) datasets^43^. The results with Bonferroni corrected p-values < 0.05 were selected. For the annotation of cancer driver genes, 138 genes were extracted from the previous study^20^.

For the analysis of trans-factor binding sites, 50 datasets of ChIP-Seq data of transcription factors, chromatin remodeling factors and RNA binding proteins were obtained from the ENCODE site (https://www.encodeproject.org/)^16^. The optical idr threshold peaks in the bed narrowPeak file (GRCh38) were downloaded and used for the analysis. To further analyze motifs that could be gained or lost by regulatory mutations, transcription factor binding motifs were searched using the Match program from the TRANSFAC database (2015.1)^25,44^. The input sequences were ±10 bp from the position of the regulatory mutations. The results with a matrix similarity score >0.95 were extracted, and candidates from a variant sequence were compared with those from a reference sequence. Sequence logos for the position weight matrix of the binding consensus were created from TRANSFAC matrices using R library seqLogo^45^.

### Survival analysis

The RNA-Seq v2 data and clinical information of TCGA lung adenocarcinoma (TCGA-LUAD) was downloaded from the NCI Genomic Data Commons using TCGA-Assembler v2.0.1^46^ (the data were downloaded on 2017/03/09). The normalized counts of genes expression (assayPlatform = gene.normalized_RNAseq) were used. Expression levels were log2 transformed after adding 1. The data of overall survival and disease-free survival duration from each case were extracted from clinical patient and follow up files. For each gene, the case group with high expression levels (more than 0.5 standard deviation from the average expression levels) and low expression levels (less than 0.5 standard deviations from the average expression levels) were defined. Kaplan-Meyer analysis with a log-rank test was conducted using the survival package in R (high expression group vs. others and low expression group vs. others).

### FANTOM CAT and FANTOM5 enhancer

FANTOM CAT lv3 robust lncRNA region (FANTOM_CAT.lv3_robust.all_lncRNA.bed.gz)^22^ and FANTOM5 phase1 and 2 permissive enhancer (human permissive enhancers phase 1 and 2.bed.gz)^23^ were taken from RIKEN database then ported to to UCSC hg38 human genome by using liftover^47^. We then checked if any of our 137 regulatory SNVs fall within region specified by the databases.

### Luciferase assay

pNL3.1 (#N1031, Promega) was selected as vector and pGL4.53 (#E5011, Promega) as control. Mutant and Wildtype fragment DNAs were inserted into pNL3.1 vector by Quick Ligation Protocol (M2200, New England Biolabs) using NheI-HF (R3131S, New England Biolabs) and HindII-HF (R3104S, New England Biolabs) according to manufacturer instructions (see Supplementary Table S10 for fragment sequences). Transformation was done using 5 Minute Transformation Protocol (C2987H/C2987I) (New England Biolabs) and plasmids were purified by PureLink™ HiPure Plasmid Kits (K2100, Thermo Fisher Scientific) according to instructions. Transfection was done by ViaFect^TM^ Transfection Reagent (E4981, Promega) according to manufacturer instructions with medium to final volume ratio of 4:1. Cells were assay after 24 hours using Nano-Glo Dual-Luciferase Reporter Assay System (N1610, Promega) according to instructions with CentroXS3 LB960 (Berthold Technology) and measurement time of 1 second for both ONE-Glo and NanoDLR.

### ChIP-qPCR

Chromatin immunoprecipitation were done using 20 μl of ETS-1 (D8O8A) Rabbit mAb (#14069, Cell Signaling Technology) as previously described^11^. After precipitation, quantitative real time PCR was done using Power SYBR Green PCR Master Mix (4367659, Applied Biosystems, Thermo Fisher Scientific) with previously reported control primers (RPS26)^48^ and primers targeting ±100 bps of the motif region (see Supplementary Table S10) on the 7900HT Fast Real-Time PCR System (Applied Biosystems) as previously described^49^. The qPCR products of Primer_F_2_123bp and Primer_R_shared were then underwent Sanger sequencing on 3730xl DNA Analyzer (Applied Biosystems) with their respective primers set.

### Data Availability

All synthetic long read sequencing data and TSS-Seq were deposited in the DDBJ with the accession number, DRA005894, DRA005903 and DRA005921. The other sequencing data were previously described and published^11^. Datasets in this paper are also provided in the database, DBTSS/DBKERO (http://dbtss.hgc.jp/; http://kero.hgc.jp/)^30^.

## Electronic supplementary material

Supplementary Figures and Tables

Supplementary Table S6

Supplementary Table S7

Supplementary Table S9

Supplementary Table S10
