# Supplementary material for: Identification of potential regulatory mutations using multi-omics analysis and haplotyping of lung adenocarcinoma cell lines
Source: Sci Rep. 2018 Mar 21;8:4926. doi: 10.1038/s41598-018-23342-1 (PMC5862974; doi:10.1038/s41598-018-23342-1)
Supplement: Supplementary file 1 — Supplementary Figures and Tables [file 41598_2018_23342_MOESM1_ESM.pdf]

Supplementary Materials

Identification of potential regulatory mutations using multi-omics analysis and haplotyping of lung adenocarcinoma cell lines

Sarun Sereewattanawoot, Ayako Suzuki, Masahide Seki, Yoshitaka Sakamoto, Takashi Kohno, Sumio Sugano, Katsuya Tsuchihara, Yutaka Suzuki

Table of Contents

Supplementary Materials Legends 2
Supplementary Figure S1 5
Supplementary Figure S2 6
Supplementary Figure S3 7
Supplementary Figure S4 8
Supplementary Figure S5 9
Supplementary Figure S6 10
Supplementary Figure S7 11
Supplementary Figure S8. 12
Supplementary Figure S9 13
Supplementary Figure S10 15
Supplementary Table S1 20
Supplementary Table S2 21
Supplementary Table S3 22
Supplementary Table S4 23
Supplementary Table S5 24
Supplementary Table S8 25

*Supplementary Table S6, Supplementary Table S7, Supplementary Table S9 and Supplementary Table S10 are provided in separate Microsoft Excel files.

Supplementary Materials Legends

Supplementary Fig. S1. Scheme of haplotype construction.

Graphic representation of haplotype construction based on the Molecular Indexes (MIs). Each circle represents one variant (ref or alt) in one SNP/SNV position. Circles that are connected horizontally are members of the same MIs (left box) or haplotypes (center and right boxes). Vertically aligned circles occupied the same SNP/SNV position in the genome. Thin arrows and rectangles indicate components of each haplotype.

Supplementary Fig. S2. Ploidy of the cell lines.

Histogram comparing the average ploidy reported by implementing the phasing strategy and the average ploidy previously reported by COSMIC. COSMIC data are available for 17 of the 23 cell line. For those cell lines, average ploidy is 3.48 – 2.73 (average 3.23) for our phasing and 3.13 - 1.99 (average 3.04) for COSMIC.

Supplementary Fig. S3. Relation between sequencing depths and phase block coverage in LC2/ad

Curves showing relation between sequencing depths and phase blocks (**A**) and phased SNPs/SNVs (**B**) in LC2/ad cell line. Blue dots represent cumulative coverage of 1-13 MinION runs, curves are logarithmic regression of the dots.

Supplementary Fig. S4. HiC analysis of regulatory mutations.

(**A, B**) Association between regulatory mutations and the promoters in topologically associating domains (TADs). The number for phased regulatory mutations (A) and phased with biased expression (**B**) are shown in the graphs. (**C**) An example of TADs with regulatory mutations. A regulatory mutation located in different TAD of the TSS is visualized in A549 HiC data using the WashU EpiGenome Browser.

Supplementary Fig. S5. PANTHER GO-Slim analysis of imprinted genes.

GO-term overrepresentation analysis by Panther using the Panther GO-Slim Biological Process database. Only "cell-cell adhesion", a subset of cellular processes (bottom) was significantly enriched (6.33 folds; Bonferroni corrected P=0.03).

Supplementary Fig. S6. Examples of allelic expression imbalances caused by imprinting.

Visualization of *MAP2K3* (**A**) and *BCLAF1* (**B**), which are transcriptionally imprinted in all cell lines regardless of the presence or expression of regulatory SNVs. The tables indicate the presence of coding region SNPs/SNVs in each cell line and blue entries mean indicate presence while white entries indicate absence. Due to the large number of cell lines and variants, only 4 common SNPs/SNVs from *MAP2K3* and 2 from *BCLAF1* are shown via the genomic viewer (IGV).

Supplementary Fig. S7. RefSeq transcripts with biased allele expression.

Breakdown of RefSeq transcripts with both regulatory SNVs ChIP-imbalance expression and coding SNPs/SNVs RNA imbalance expression, partition by chromosome X and other chromosomes.

Supplementary Fig S8. Haplotyping of PDGFRA gene in H1703 cell line

Each character denotes nucleotide in each haplotype's SNPs/SNVs position; (+) indicates insertion after that position and (-) indicates deletion after that position.

Supplementary Fig. S9. Gain/loss of regulatory elements by regulatory mutations.

(**A**) A regulatory mutation of *ZNF594* in A427 cell line. A promoter mutation and three coding SNPs are shown with IGV visualization of whole-genome sequencing and RNA-seq data (upper). 18 ENCODE ChIP-Seq peaks were overlapped with the regulatory mutation (lower panel). (**B**) A regulatory mutation of *ERAP2* in H1975 cell line. A promoter mutation and coding SNV are shown using IGV. (**C**) The *RNF2* binding peak in ENCODE ChIP-Seq data overlapped with a regulatory mutation of *NFATC1* in RERF-LC-Ad1 (left). Expression levels of *NFATC1* in the cell lines are shown in the right.

Supplementary Fig. S10. Survival analysis of 31 genes with regulatory mutations using TCGA-LUAD data.

Kaplan-Meier analysis of cases in TCGA-LUAD data divided into two groups depending on expression levels of the genes with regulatory mutations. (**A, B**) The cases with high expression levels of a given gene significantly showed good or poor prognosis comparing with the other cases. (**C, D**) The cases with low expression levels of a given gene significantly showed good or poor prognosis comparing with the other cases.

Supplementary Table S1.Summary of SNPs/SNVs detected by Illumina short-read sequencing.

Genic SNPs/SNVs are those in the exon, intron and UTR. Coding SNPs/SNVs are those only in exons. Regulatory SNVs are those in “peak” region of each ChiP marker. Each Chip-Seq marker dataset was counted separately.

Supplementary Table S2. Detailed statistics of implemented phasing scheme.

Detailed statistics of the 10x synthetic long-read sequencing with phasing statistics from the implemented phasing scheme (the original 10x Long Ranger results are summarized in Table 1). Block lengths were calculated by subtracting the distance between the most 5’ SNP/SNV and the most 3’ SNP/SNV in each phase block.

Supplementary Table S3. Statistics of the MinION physical long read sequencing.

We performed nine 2D runs and one 1D run for H1975, three 2D runs for RERF-LC-KJ and three 1D and 10 $1D^2$ runs for LC/2ad. For the 2D runs, only paired read that passed MinION’s quality control were used. For the 1D and $1D^2$ run, any read that passed quality control was used. Reads from every run were then merged and analyzed as a single dataset.

Supplementary Table S4. Validation analysis of the phasing results by MinION reads.

Summary of validation analysis by MinION. Only reads with >10 mapping quality were considered. Blocks that had at least twice supportive reads were considered validated.

For 1D and 1D^2 runs (LC2ad), only nucleotides with base call quality over 15 were considered.

Supplementary Table S5. The number of heterozygous SNVs with imbalanced and balanced transcriptions.

The number of SNVs called and considered heterozygous by GATK HaplotypeCaller categorized by chromosome and expression pattern**.**

Supplementary Table S6. A list of imprinted RefSeq transcripts in more than 7 cell lines.

This table is provided in separate Excel file.

Supplementary Table S7. A list of regulatory mutations phased and allelic-biased in the 23 cell lines.

The full table is provided in separate Excel file.

Supplementary Table S8. PANTHER GO-Slim overrepresentation analysis of regulatory SNVs.

Only GO-Terms with Benjamini P-values < 0.05 are shown. “DNA binding” (GO:0003677)

, “Regulation of gene expression, epigenetic” (GO:0040029) and “Regulation of nucleobase-containing compound metabolic process” (GO:0019219) are overrepresented, hinting a more complex downstream effects of those regulatory mutations.

Supplementary Table S9 Lists of regulatory SNVs associated with lncRNA and enhancer regions in the FANTOM5 database

The table is provided by the separate excel file.

(A) Regulatory SNVs associated with FANTOM CAT

(B) Regulatory SNVs associated with enhancer regions

Supplementary Table S10 Sequences of DNA fragments and primers for validation experiments

The table is provided by the separate excel file.

(A) DNA fragments used for Luciferase assay

(B) Primers used for qPCR

Supplementary Figure S1 Scheme of haplotype construction

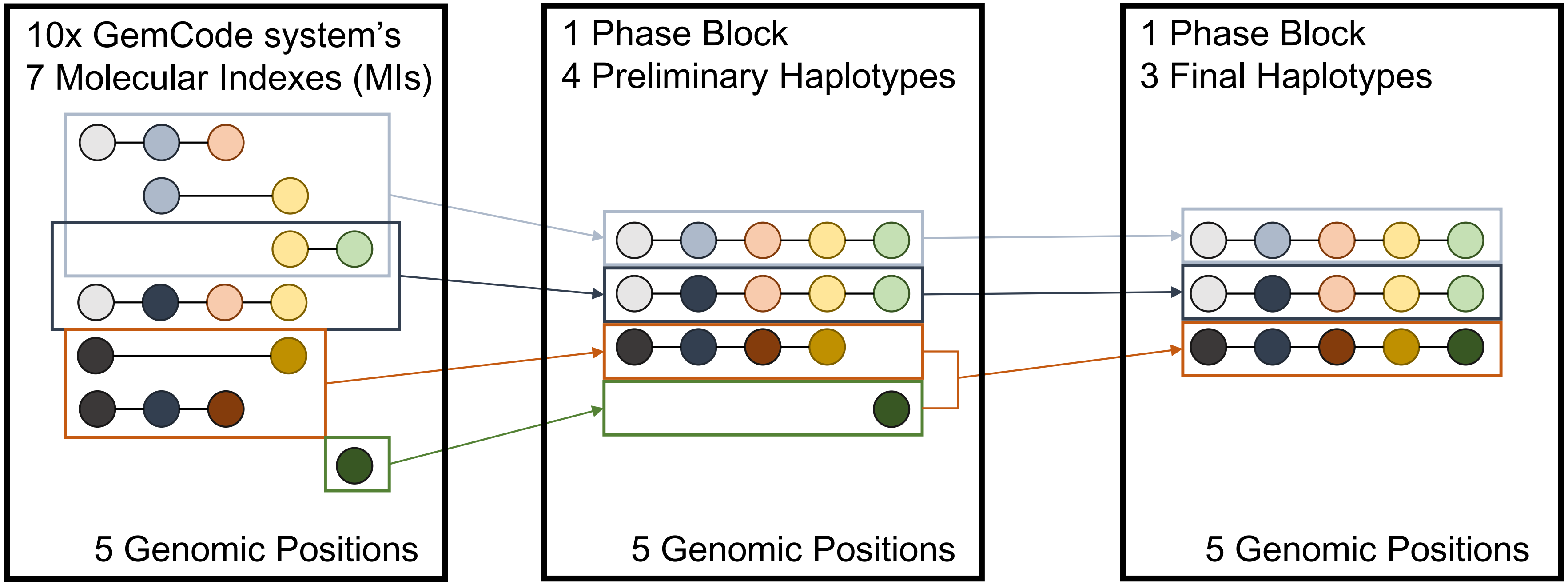

Supplementary Figure S2 Ploidy of the cell lines

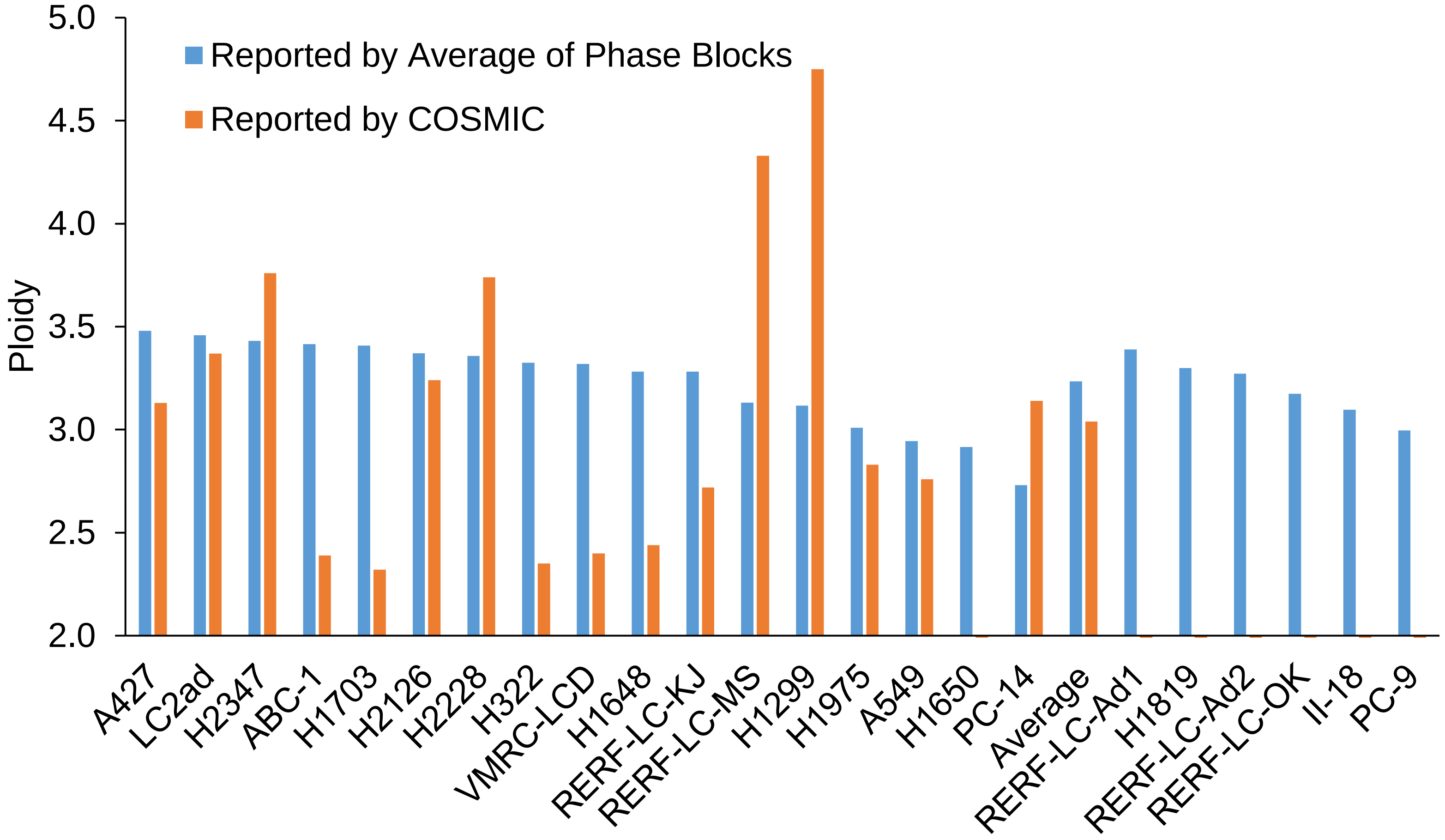

Supplementary Figure S3 Relation between sequencing depths and phase block coverage in LC2/ad

A

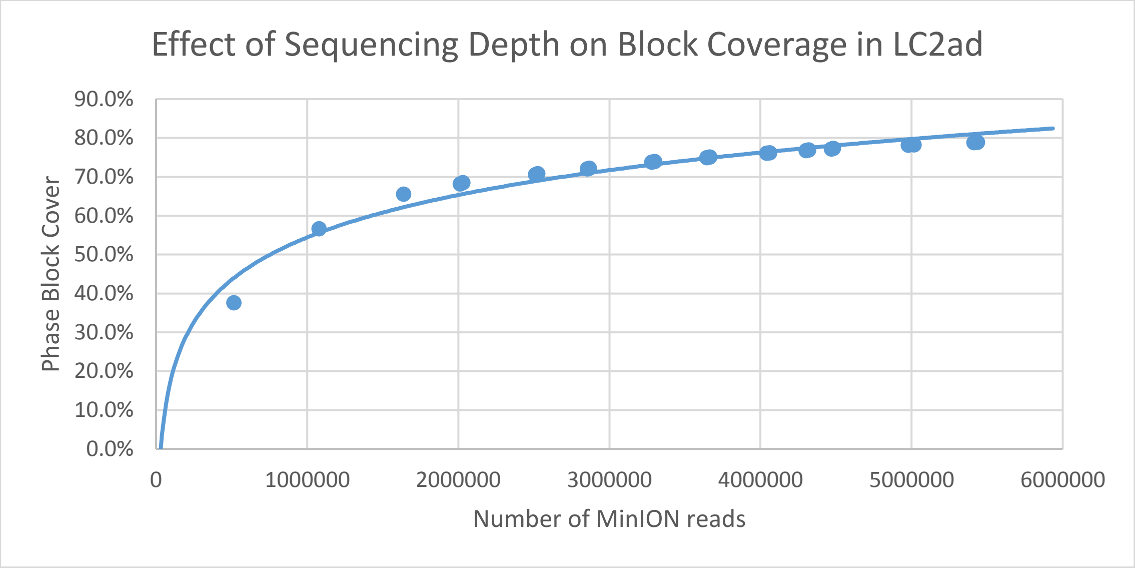

B

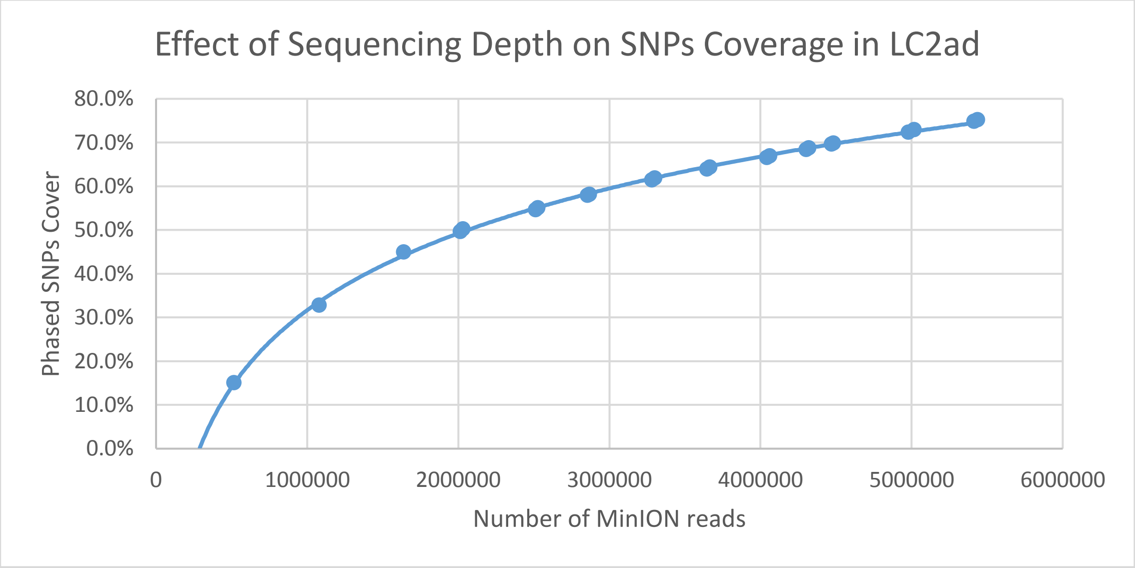

Supplementary Figure S4 HiC analysis of regulatory mutations

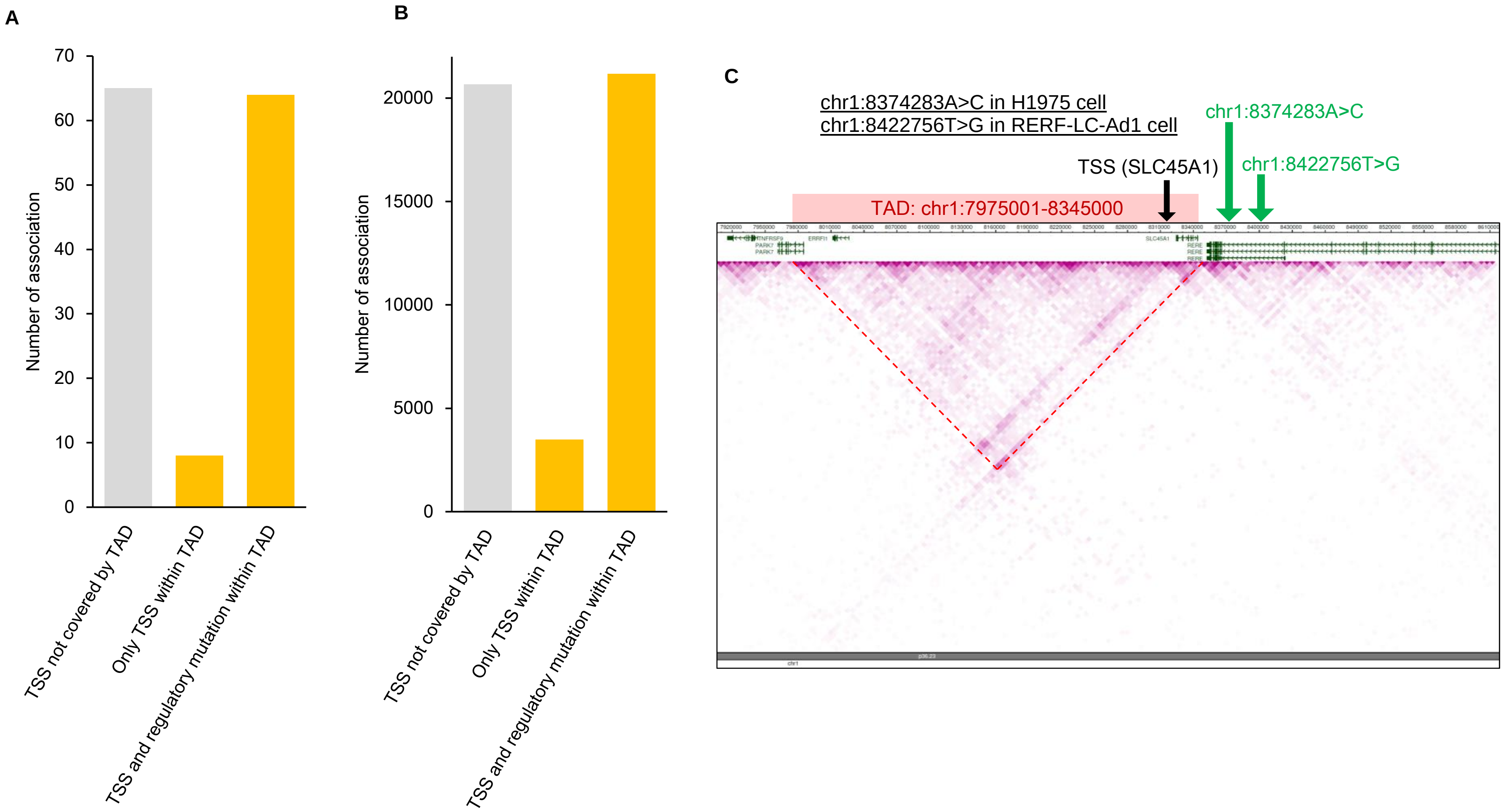

Supplementary Figure S5 PANTHER GO-Slim analysis of imprinted genes

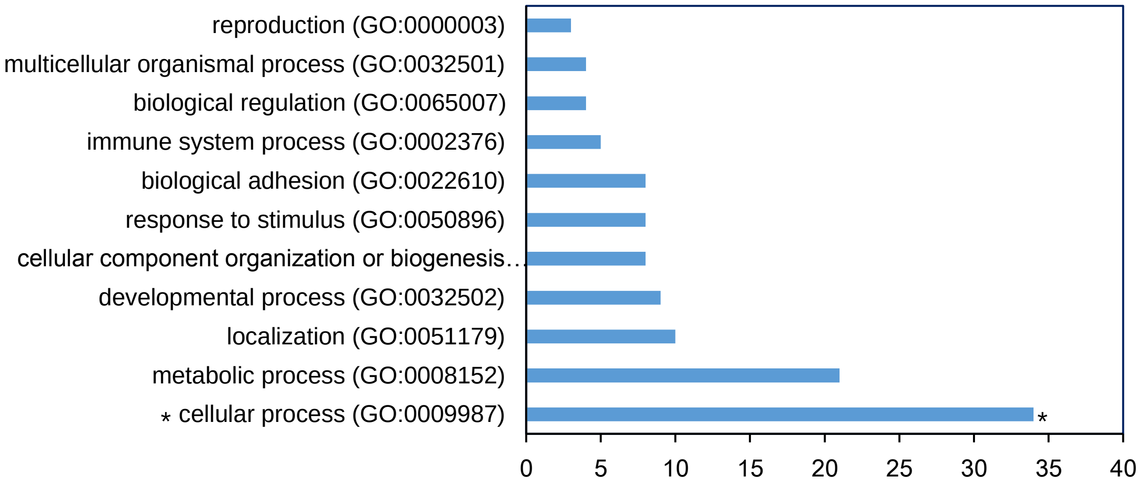

*"cell-cell adhesion" (GO:0016337), a subset of cellular process, is significantly enriched (6.33 folds; Benjamini P= 0.03)

Supplementary Figure S6 Examples of allelic expression imbalances caused by imprinting

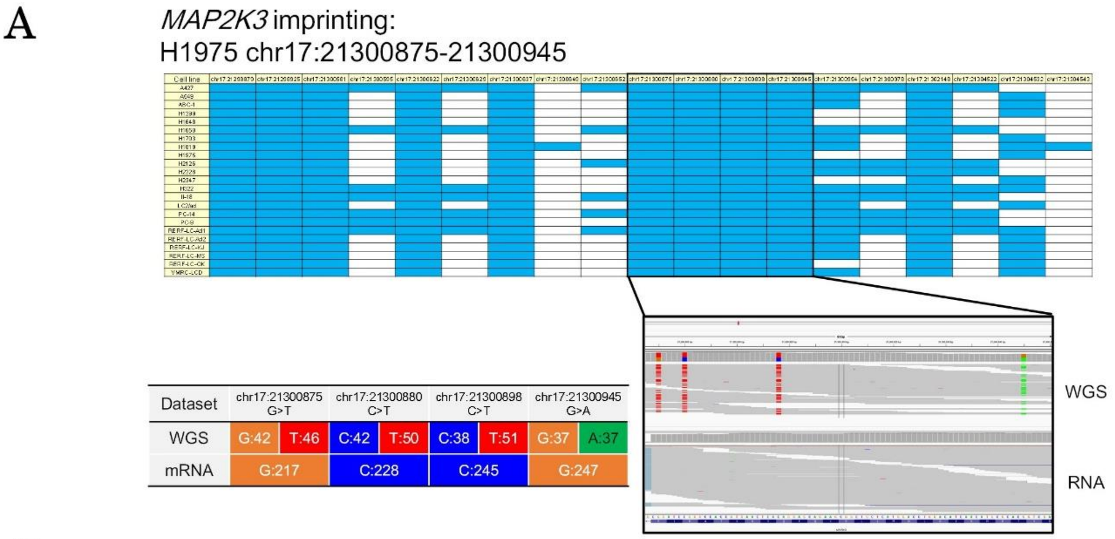

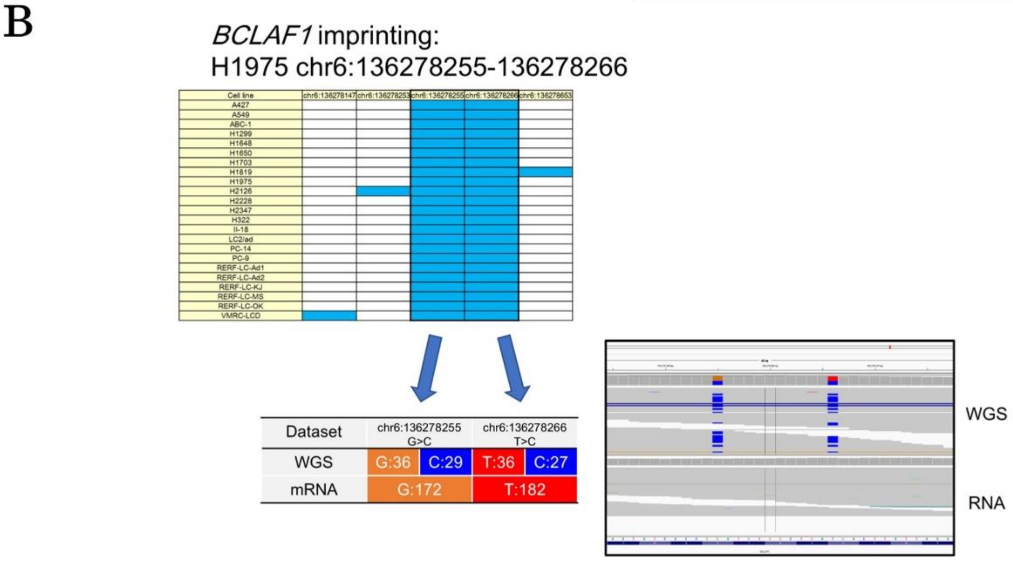

Supplementary Figure S7 RefSeq transcripts with biased allele expression

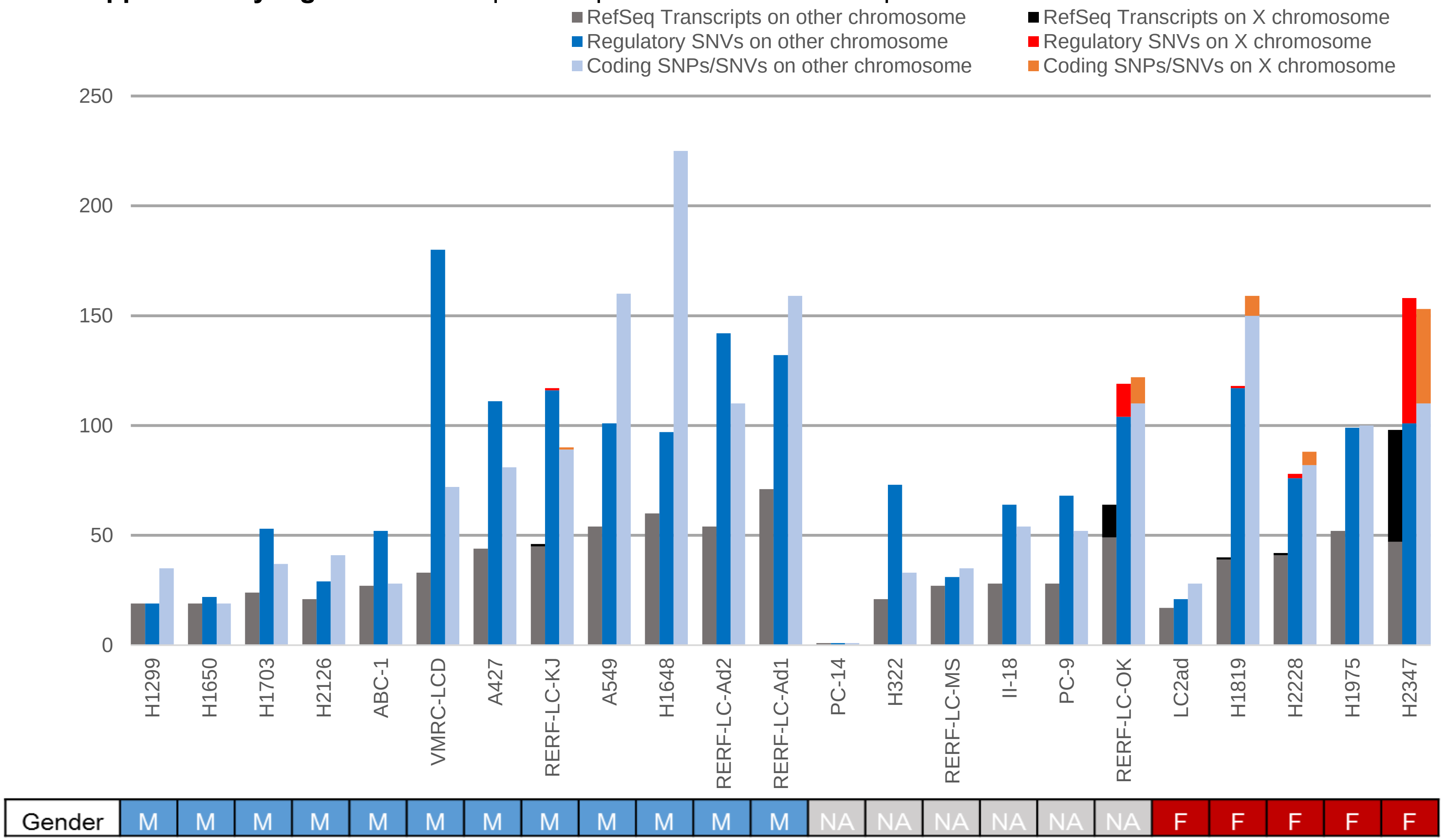

Supplementary Figure S8. Haplotyping of PDGFRA gene in H1703 cell line

Regulatory SNVs

Coding SNPs

Haplotype Number	chr4:54221414	chr4:54222376	chr4:54222594	chr4:54222742	chr4:54226459	chr4:54227622	chr4:54227747	chr4:54227788	chr4:54227864	chr4:54228300	chr4:54228462	chr4:54230013	chr4:54232874	chr4:54236258	chr4:54240528	chr4:54240616	chr4:54258424	chr4:54263911
1	A	T	T	A	C	(-)AACTT	G	A	G	A	T	A	A	C	C	C	G	C
2	A	T	T	A	C	(-)AACTT	G	A	G	A	T	A	A	C	C	C	G	C
3	A	T	T	A	C	(-)AACTT	G	A	G	A	T	A	A	C	C	C	G	C
4	A	T	T	A	C	(-)AACTT	G	A	G	A	T	A	A	C	C	C	G	C
5	A	C	T	A	C	(-)AACTT	A	A	G	A	T	A	A	C	T	G	A	T
6	A	C	C	A	T	C	A	G	C	G	G	A	A	C	T	G	A	T
7	A	C	C	A	T	C	A	G	C	G	G	A	A	C	T	G	A	T
8	A	C	C	A	T	C	A	G	C	G	G	A	A	C	T	G	A	T
9	C	T	C	C	T	C	A	G	C	G	G	T	C	T	T	G	A	T

Haplotype Number	chr4:54263987	chr4:54264627	chr4:54264834	chr4:54265283	chr4:54267559	chr4:54267769	chr4:54267792	chr4:54271016	chr4:54271934	chr4:54273604	chr4:54273849	chr4:54275126	chr4:54275333	chr4:54275365	chr4:54277410	chr4:54277854	chr4:54278223
1	A	G	(-)A	A	G	T	T	C	C	C	T	A	T	T	A	T	T
2	A	G	(-)A	A	G	T	T	C	C	C	T	A	T	T	A	T	T
3	A	G	(-)A	A	G	T	T	C	(+)TCCT	C	T	A	T	T	A	T	T
4	A	G	T	A	G	T	T	C	(+)TCCT	C	T	A	T	T	A	T	(-)AAA
5	G	T	T	C	T	C	C	T	C	T	C	G	C	A	G	T	T
6	G	T	T	C	T	C	C	T	C	T	C	G	C	A	G	G	T
7	G	T	T	C	T	C	C	T	C	T	C	G	C	A	G	G	T
8	G	T	T	C	T	C	C	T	C	T	C	G	C	A	G	G	T
9	G	T	T	C	T	C	C	T	C	T	C	G	C	A	G	G	T

Haplotype Number	chr4:54278246	chr4:54279091	chr4:54280222	chr4:54280239	chr4:54280587	chr4:54280759	chr4:54280760	chr4:54284564	chr4:54285791	chr4:54285836	chr4:54285873	chr4:54286117	chr4:54286319	chr4:54287740	chr4:54287942	chr4:54288360	chr4:54288724
1	A	G	T	T	A	T	T	(+)AG	T	T	T	T	(-)TTTATTT ATTATTTA	C	T	C	C
2	A	G	T	(+)TGTAGGT CCCCCAG	A	T	T	(+)AG	T	T	T	T	(-)TTTATTT ATTATTTA	C	T	C	C
3	C	G	T	(+)TGTAGGT CCCCCAG	A	T	T	(+)AG	T	T	T	T	(-)TTTATTT ATTATTTA	C	T	C	C
4	C	G	T	(+)TGTAGGT CCCCCAG	A	T	T	(+)AG	T	T	T	T	(-)TTTATTT ATTATTTA	C	T	C	C
5	A	A	C	T	C	A	A	C	(+)A	G	C	G	T	T	C	G	T
6	A	A	C	(+)TGTAGGT CCCCCAG	C	A	A	C	(+)A	G	C	G	T	T	C	G	T
7	A	A	C	(+)TGTAGGT CCCCCAG	A	A	A	C	(+)A	G	C	G	T	T	C	G	T
8	A	A	C	T	C	A	A	C	(+)A	G	C	G	T	T	C	G	T
9	A	A	C	(+)TGTAGGT CCCCCAG	C	A	A	C	(+)A	G	C	G	T	T	C	G	T

Supplementary Figure S9 Gain/loss of regulatory elements by regulatory mutations

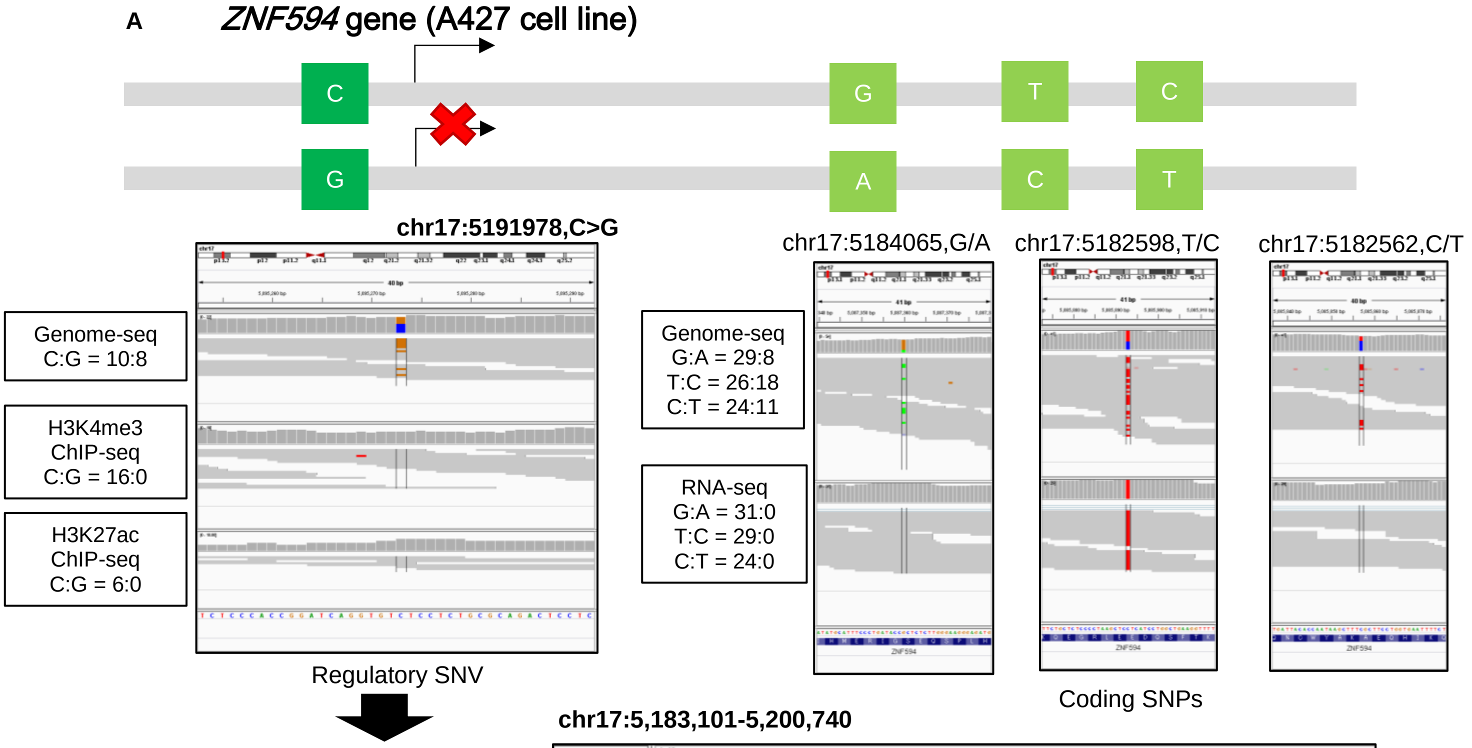

ENCODE ChIP-seq	File ID (bed narrowPeak)
CTCF	ENCFF646TUX
CTCF	ENCFF535MZG
ELF1_EtOH	ENCFF935ZUW
ETS1_EtOH	ENCFF896WFR
GABPA_EtOH	ENCFF520GJC
MYC	ENCFF542GMN
NR3C1_DEX50nM	ENCFF114SRD
NR3C1_EtOH	ENCFF463DJO
POLR2A_DEX100nM	ENCFF915LKZ
POLR2A_EtOH	ENCFF664KTN
RAD21	ENCFF897QCA
SIN3A	ENCFF567BJI
SIX5_EtOH	ENCFF189NMX
SMC3	ENCFF256LDD
SP1_EtOH	ENCFF404OSB
TAF1_EtOH	ENCFF886KDK
TCF12_EtOH	ENCFF228CDD
ZBTB33_EtOH	ENCFF593ZJA

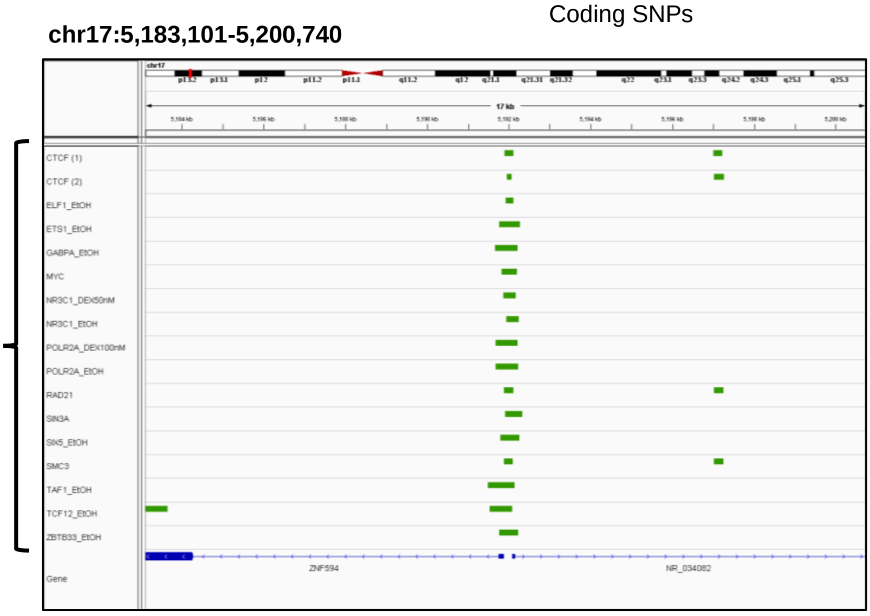

B

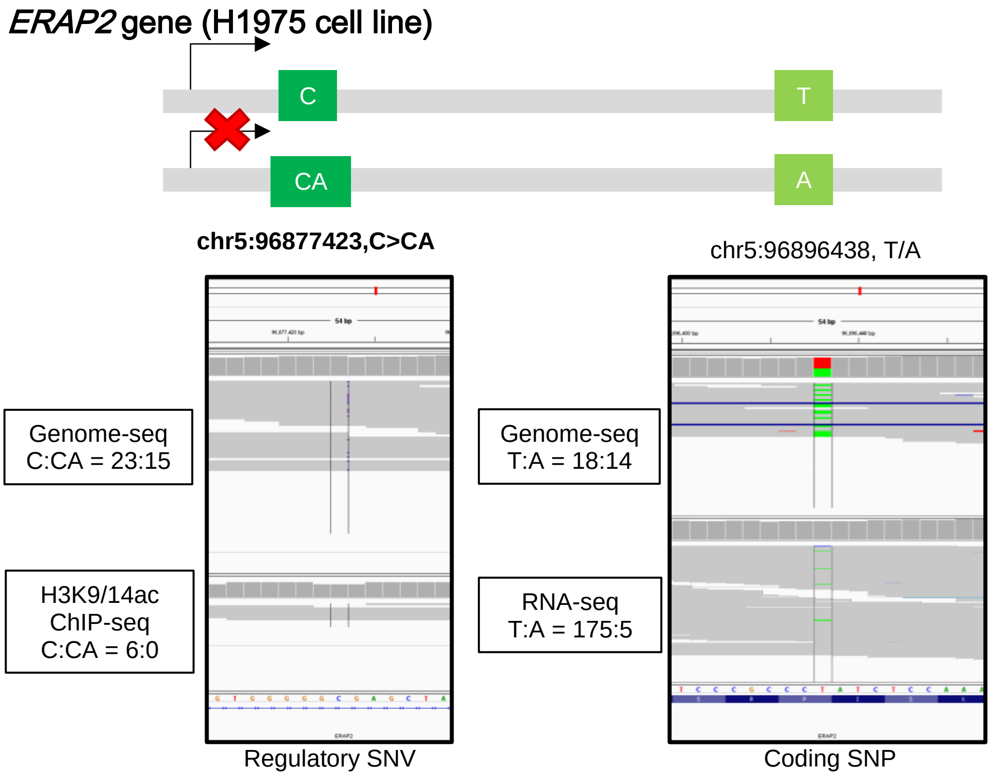
ERAP2 gene (H1975 cell line)
C
T
CA
A
chr5:96877423,C>CA
chr5:96896438, T/A
Genome-seq
C:CA = 23:15
H3K9/14ac
ChIP-seq
C:CA = 6:0
Genome-seq
T:A = 18:14
RNA-seq
T:A = 175:5
Regulatory SNV
Coding SNP

C

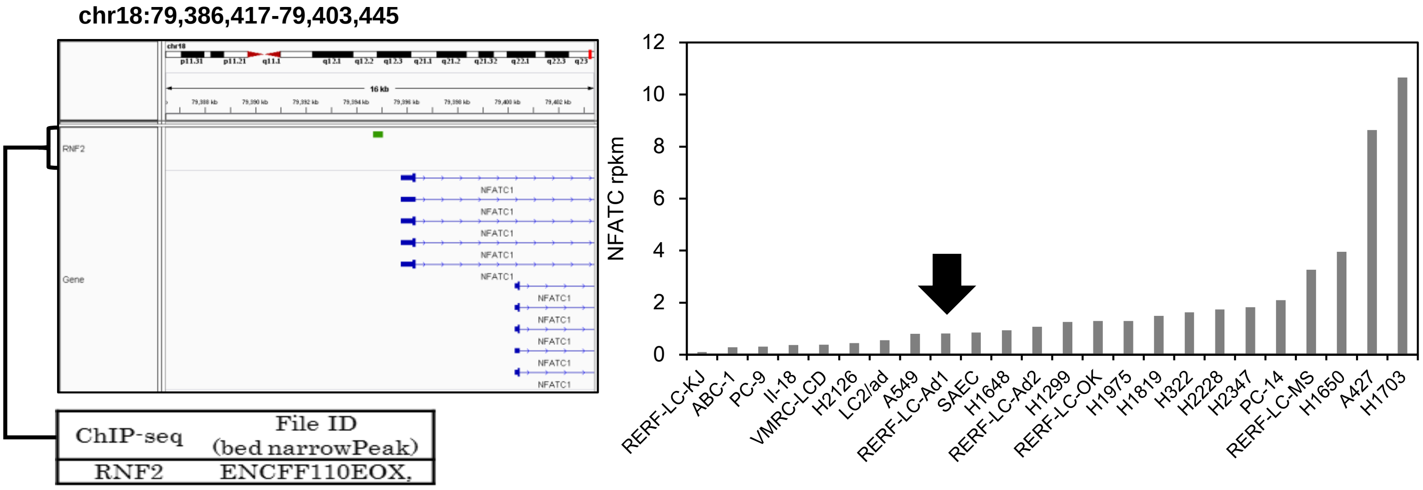
chr18:79,386,417-79,403,445
RNF2
Gene
NFATC1
ChIP-seq
File ID
(bed narrowPeak)
RNF2
ENCFF110EOX,
NFATC rpkm
0
2
4
6
8
10
12
RERF-LC-KJ
ABC-1
PC-9
II-18
VMRC-LCD
H2126
LC2/ad
A549
RERF-LC-Ad1
SAEC
H1648
RERF-LC-Ad2
H1299
RERF-LC-OK
H1975
H1819
H322
H2228
H2347
PC-14
RERF-LC-MS
H1650
A427
H1703

Supplementary Figure S10 Survival analysis of 31 genes with regulatory mutations using TCGA-LUAD data

A

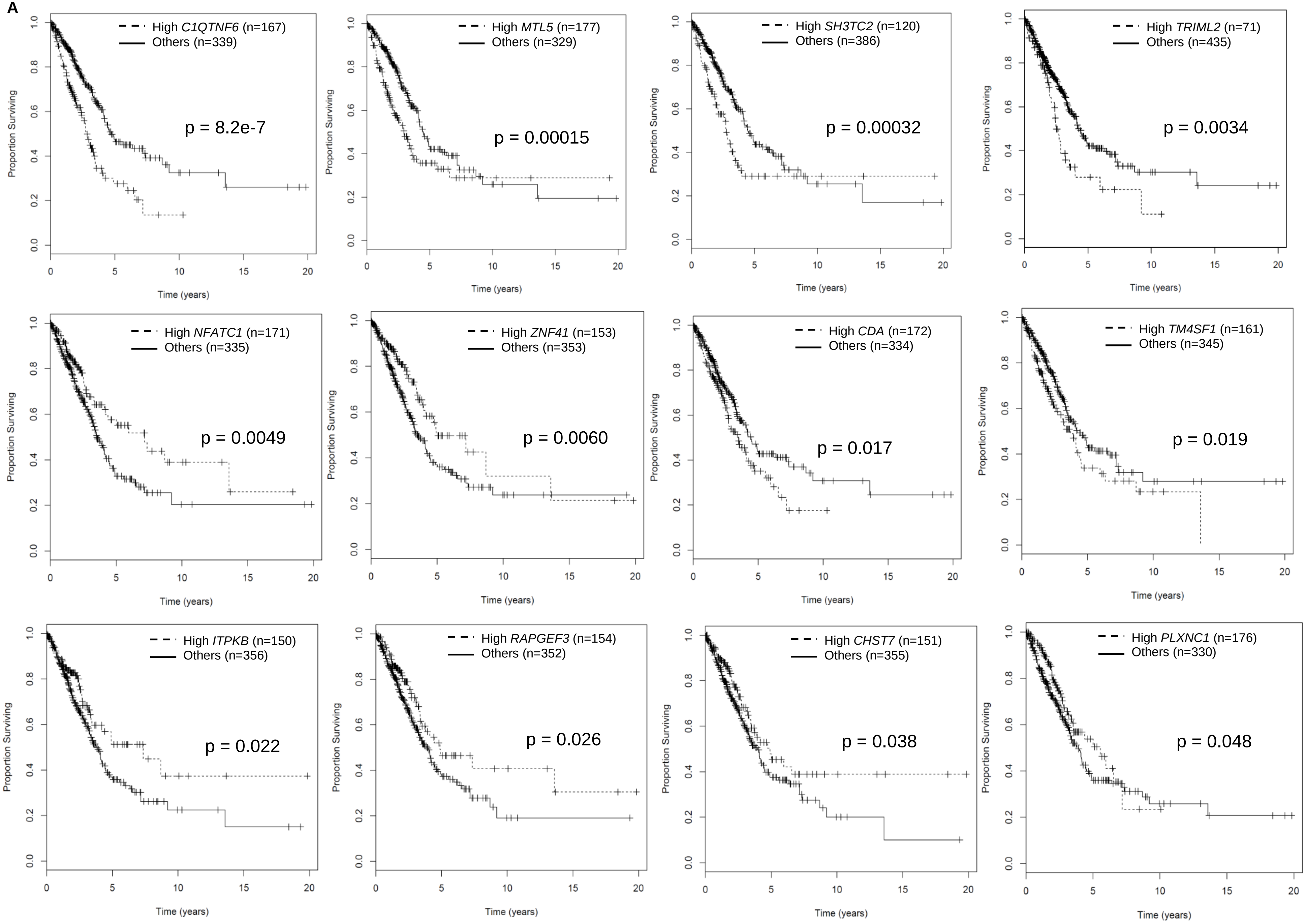

B

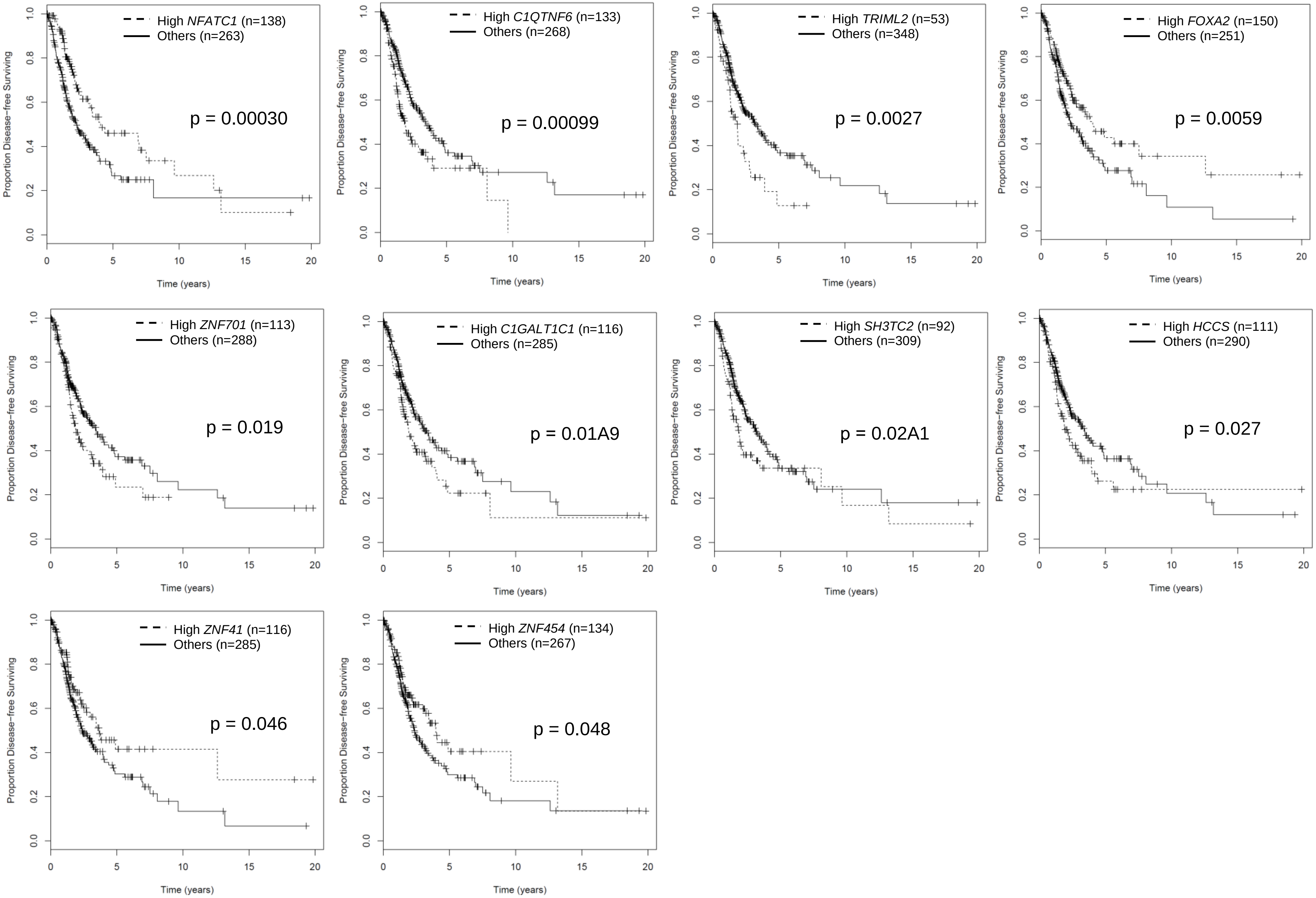

High NFATC1 (n=138)
Others (n=263)
p = 0.00030
High C1QTNF6 (n=133)
Others (n=268)
p = 0.00099
High TRIML2 (n=53)
Others (n=348)
p = 0.0027
High FOXA2 (n=150)
Others (n=251)
p = 0.0059
High ZNF701 (n=113)
Others (n=288)
p = 0.019
High C1GALT1C1 (n=116)
Others (n=285)
p = 0.01A9
High SH3TC2 (n=92)
Others (n=309)
p = 0.02A1
High HCCS (n=111)
Others (n=290)
p = 0.027
High ZNF41 (n=116)
Others (n=285)
p = 0.046
High ZNF454 (n=134)
Others (n=267)
p = 0.048
Proportion Disease−free Surviving
Time (years)

C

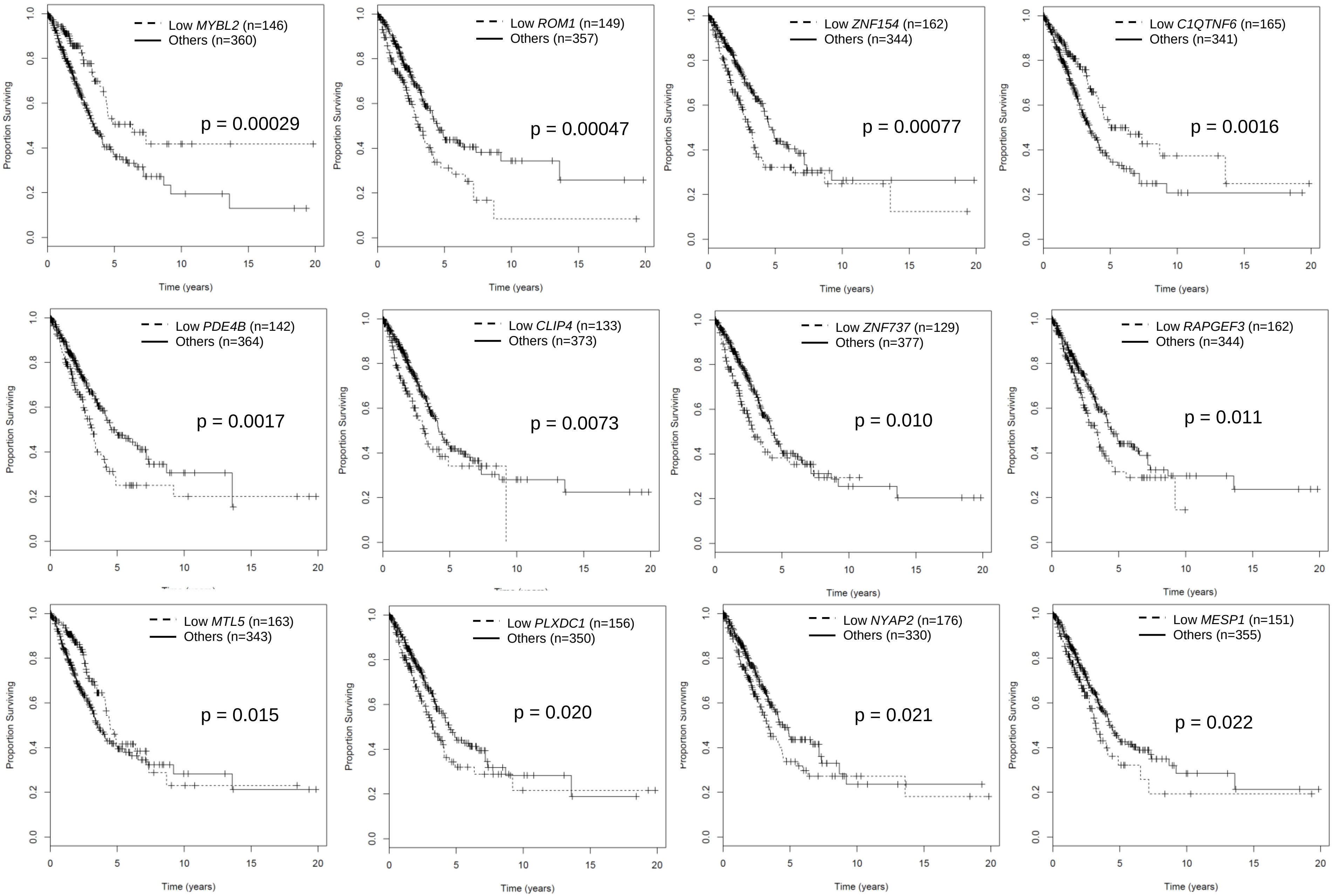
Low MYBL2 (n=146)
Others (n=360)
p = 0.00029
Low ROM1 (n=149)
Others (n=357)
p = 0.00047
Low ZNF154 (n=162)
Others (n=344)
p = 0.00077
Low C1QTNF6 (n=165)
Others (n=341)
p = 0.0016
Low PDE4B (n=142)
Others (n=364)
p = 0.0017
Low CLIP4 (n=133)
Others (n=373)
p = 0.0073
Low ZNF737 (n=129)
Others (n=377)
p = 0.010
Low RAPGEF3 (n=162)
Others (n=344)
p = 0.011
Low MTL5 (n=163)
Others (n=343)
p = 0.015
Low PLXDC1 (n=156)
Others (n=350)
p = 0.020
Low NYAP2 (n=176)
Others (n=330)
p = 0.021
Low MESP1 (n=151)
Others (n=355)
p = 0.022
Proportion Surviving
Time (years)

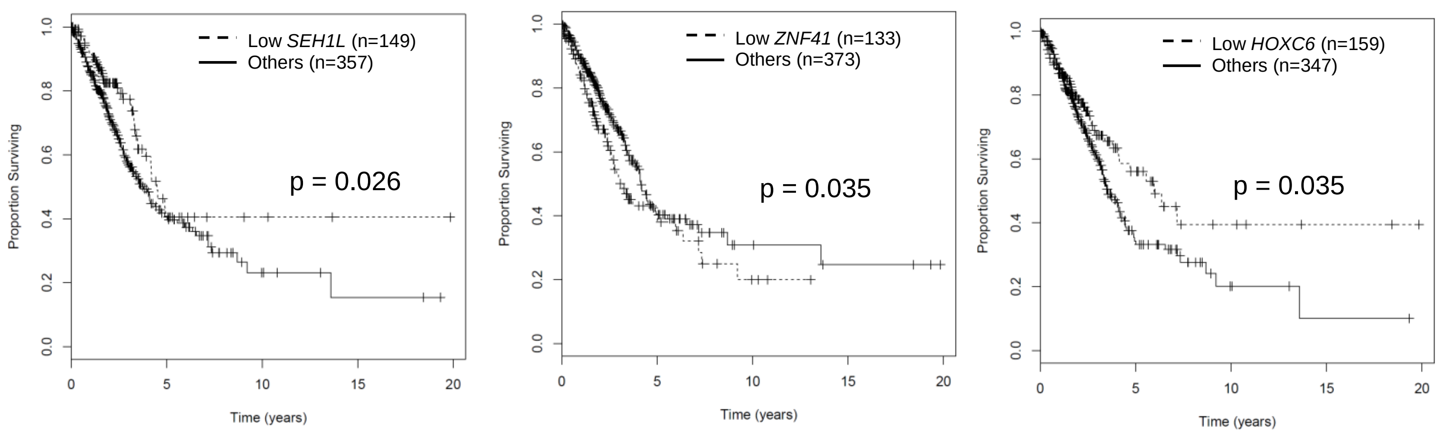
Low SEH1L (n=149)
Others (n=357)
p = 0.026
Proportion Surviving
Time (years)
Low ZNF41 (n=133)
Others (n=373)
p = 0.035
Proportion Surviving
Time (years)
Low HOXC6 (n=159)
Others (n=347)
p = 0.035
Proportion Surviving
Time (years)

D

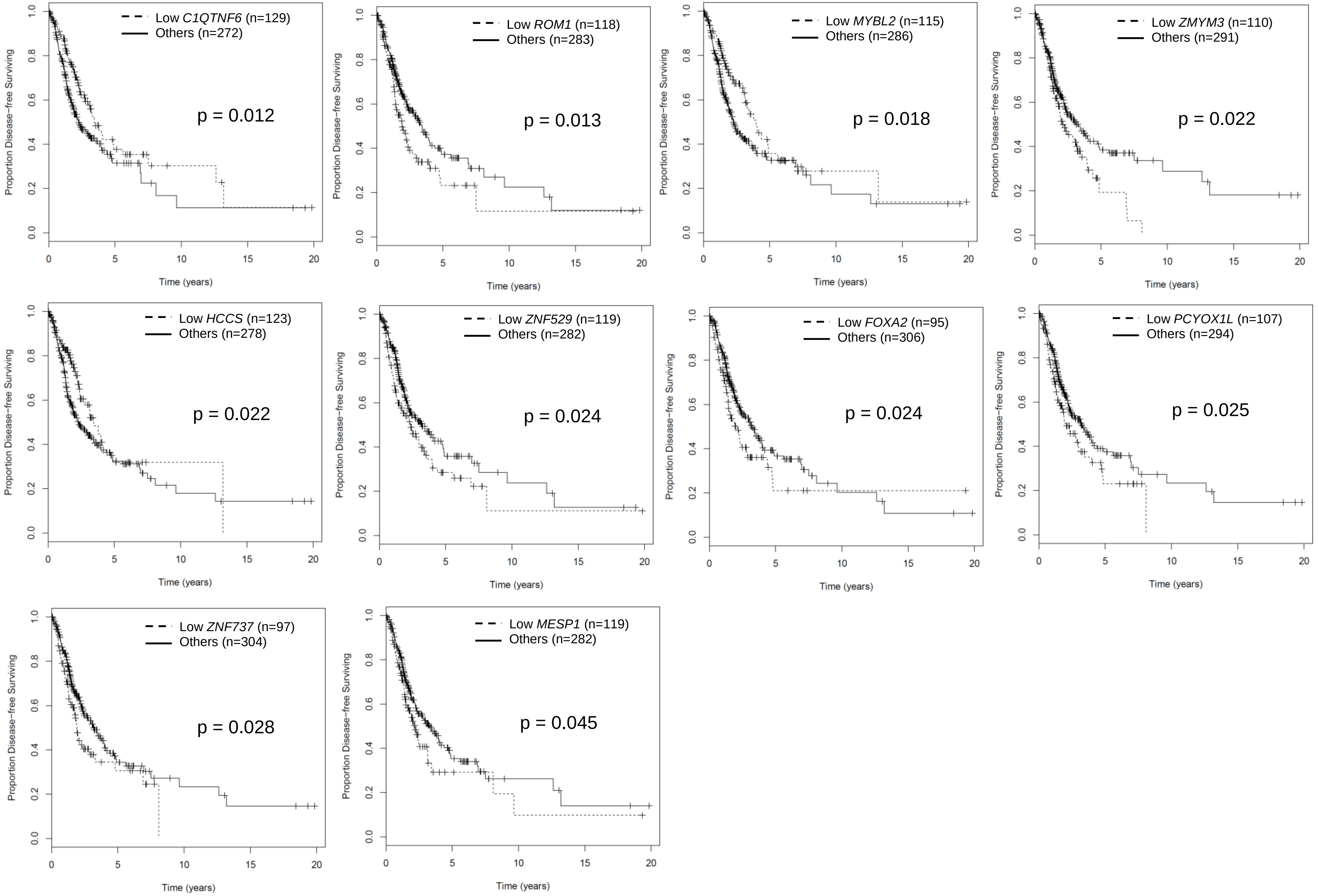
Low C1QTNF6 (n=129)
Others (n=272)
p = 0.012
Low ROM1 (n=118)
Others (n=283)
p = 0.013
Low MYBL2 (n=115)
Others (n=286)
p = 0.018
Low ZMYM3 (n=110)
Others (n=291)
p = 0.022
Low HCCS (n=123)
Others (n=278)
p = 0.022
Low ZNF529 (n=119)
Others (n=282)
p = 0.024
Low FOXA2 (n=95)
Others (n=306)
p = 0.024
Low PCYOX1L (n=107)
Others (n=294)
p = 0.025
Low ZNF737 (n=97)
Others (n=304)
p = 0.028
Low MESP1 (n=119)
Others (n=282)
p = 0.045
Proportion Disease−free Surviving
Time (years)

Supplementary Table S1 Summary of SNPs/SNVs detected by Illumina short-read sequencing

Cell line	Whole-genome sequencing			SNPs/SNVs from GATK			
	Mapped reads	%mapped reads	Depths	Raw SNPs/SNVs	Genic SNPs/SNVs	Coding SNPs/SNVs	Regulatory SNVs
A427	1,084,672,075	94%	34.6	4,024,063	1,397,615	18,775	70,651
A549	762,328,900	71%	22.7	4,228,434	1,150,303	17,890	46,197
ABC-1	1,198,942,503	94%	38.4	3,918,935	1,359,715	18,666	15,685
H322	921,462,662	95%	29.1	3,710,129	1,273,472	17,904	20,267
H1299	930,092,532	95%	29.9	3,910,954	1,343,074	18,287	49,532
H1648	1,129,800,194	94%	38.0	4,842,219	1,668,060	27,552	53,569
H1650	1,093,147,187	96%	35.0	3,738,924	1,272,227	17,280	68,423
H1703	1,035,232,011	87%	31.9	3,908,849	1,340,392	18,276	48,255
H1819	1,197,312,856	92%	38.1	4,169,230	1,441,883	19,326	65,711
H1975	1,056,952,131	94%	33.4	4,026,746	1,333,864	19,389	75,912
H2126	668,355,912	88%	21.3	4,233,027	1,457,113	19,789	36,050
H2228	855,605,013	90%	27.4	4,407,002	1,512,216	19,312	76,000
H2347	983,271,902	85%	31.6	3,265,345	1,316,041	18,102	82,687
II-18	890,312,525	84%	26.8	4,122,525	1,428,765	20,231	37,516
LC2ad	1,400,218,662	93%	44.8	3,955,271	1,372,090	18,855	35,557
PC-9	1,326,079,008	94%	42.4	3,949,215	1,368,717	18,717	42,801
PC-14	979,278,917	97%	31.3	3,712,268	1,259,609	17,977	10,460
RERF-LC-Ad1	1,265,604,463	95%	40.6	4,368,425	1,514,733	20,936	68,740
RERF-LC-Ad2	1,284,008,781	95%	41.1	4,213,008	1,449,905	19,887	69,823
RERF-LC-KJ	1,113,739,330	95%	35.6	4,135,667	1,426,828	19,961	33,029
RERF-LC-MS	1,319,743,295	93%	42.3	3,949,142	1,348,821	17,980	48,221
VMRC-LCD	1,394,724,167	93%	44.6	4,078,677	1,383,592	19,613	36,608
RERF-LC-OK	684,830,042	86%	21.0	4,011,742	1,333,768	18,749	42,278
Average	1,068,509,351	91%	34.4	4,038,252	1,380,557	19,281	49,303

Supplementary Table S2 Detailed statistics of implemented phasing scheme

10x WES + Regulatory Region (bait: 113.7 Mb)											
	Sequencing Statistics						Phasing Statistics				
Cell Line	Number of Reads	Mapped Read%	PCR Duplication	Bait Coverage	Depths	Eligible Heterozygous SNPs (WGS)	SNPs Phased	Number of Phase Blocks	Average Block Length	Longest Block Length
A427	99,593,100	99.5%	3.01%	99.4%	59.65	1,975,179	5.74%	6,986	70,717	1,213,123
A549	95,848,264	99.5%	3.21%	99.3%	56.27	1,192,617	6.31%	7,074	38,458	921,645
ABC-1	94,462,990	99.4%	17.60%	99.0%	52.33	1,600,165	5.34%	6,415	59,274	1,700,788
H322	88,136,374	99.5%	3.56%	99.1%	51.35	1,302,262	5.73%	5,378	57,835	1,240,285
H1299	103,133,700	99.4%	5.63%	99.4%	61.15	1,616,105	5.38%	7,287	47,072	1,192,363
H1648	85,929,520	99.5%	3.46%	99.4%	51.49	2,559,456	4.66%	8,051	58,395	1,521,176
H1650	85,269,994	99.5%	5.59%	99.0%	50.05	1,322,760	4.38%	5,131	41,893	1,238,886
H1703	97,084,096	99.4%	5.52%	99.3%	54.65	1,667,112	6.02%	7,055	57,862	921,997
H1819	93,562,794	99.3%	6.80%	99.2%	52.51	1,928,801	5.45%	7,578	50,257	889,804
H1975	83,093,898	99.2%	2.63%	99.1%	48.99	2,265,683	4.96%	9,382	42,142	1,377,795
H2126	95,109,618	99.4%	7.52%	99.3%	53.93	1,466,115	5.44%	5,397	70,279	1,622,928
H2228	91,567,448	99.2%	3.15%	99.4%	54.40	2,318,737	5.59%	7,958	73,144	1,501,979
H2347	93,224,434	99.4%	8.65%	99.3%	53.37	2,521,416	5.48%	9,319	60,363	1,316,657
II-18	85,938,160	99.5%	1.75%	99.1%	50.97	1,378,873	5.43%	6,658	38,159	730,910
LC2ad	87,391,948	99.1%	3.19%	99.3%	51.01	2,065,496	5.43%	6,732	79,515	1,689,253
PC-9	93,671,674	99.1%	8.50%	98.9%	55.43	1,726,066	4.93%	6,374	54,957	1,186,613
PC-14	85,912,630	99.5%	2.15%	99.3%	51.62	1,270,056	0.59%	1,342	23,491	383,302
RERF-LC-Ad1	95,459,772	99.5%	3.49%	99.3%	55.92	2,393,906	5.69%	9,244	57,500	1,245,677
RERF-LC-Ad2	85,929,050	99.5%	3.56%	99.4%	51.22	2,112,023	5.21%	7,614	59,455	1,041,153
RERF-LC-KJ	102,867,672	99.4%	5.20%	99.5%	60.16	1,963,388	5.94%	8,623	49,086	993,296
RERF-LC-MS	73,659,054	99.4%	4.91%	99.1%	41.65	1,677,568	4.11%	5,648	59,231	1,208,661
VMRC-LCD	83,375,866	99.4%	5.12%	99.1%	47.48	2,017,028	5.21%	7,868	51,510	1,530,527
RERF-LC-OK	101,048,218	99.5%	3.86%	99.4%	60.36	1,621,744	6.30%	7,981	49,848	876,013
Average	91,359,577	99.4%	5.1%	99.2%	53	1,824,459	5.18%	7,004	54,367	1,197,601

Supplementary Table S3 Statistics of the MinION physical long read sequencing used in validation of the phase blocks.

(A) H1975 and RERF-LC-KJ (R9 or R9.4 flow cell)

Cell	Run	1D read		2D read		Total*	Un-mapped	Mapped to human genome	Avg. depth	Coverage (≥1×)	Read length	
		pass	fail	pass	fail						Mean	Max
H1975	10	42,629	291	640,277	61,363	682,209	7,876	674,333 (98.8%)	0.7	0.46	4,815	179,616
RERF-LC-KJ	3	-	-	477,280	42,680	519,960	7,978	511,982 (98.5%)	0.58	0.36	3,627	118,237

*For the mapping and further analyses, we used 2D passed reads on 9 runs and 1D reads on 1 run in H1975 and 2D reads on 3 runs in RERF-LC-KJ.

(B) LC2/ad (R9.5 flow cell)

Cell	Run	Total* (1D + 1D square)	Un-mapped	Mapped to human genome	Avg. depth	Coverage (≥1×)	Read length	
							Mean	Max
LC2/ad	13	6,704,709	1,084,394	5,620,315 (83.8%)	6.6	0.93	6,572	2,495,160

*We used both 1D and 1D Square reads for the analysis.

Supplementary Table S4 Validation analysis of the phasing results by MinION reads

Cell line	H1975	RERF-LC-KJ	LC2/ad
Flow cell version	R9 + R9.4	R9.4	R9.5
Run	9 (2D passed) + 1 (1D)	3 (2D)	3 (1D) + 10 (1D square)
Phase block	9382	8623	6697
Block covered	5763	4046	5282
%block covered	61.4	46.9	78.9
SNPs in block	199,987	193,853	218,892
SNPs covered	74,916	44,018	164,656
%SNPs covered	37.5	22.7	75.2
Supported block	4963	3473	4422
Not supported block	800	573	1260
%supported block	86.1	85.8	77.8

Supplementary Table S5 The number of heterozygous SNVs with imbalanced and balanced transcriptions

Gender	Cell line	Autosome + Y				X			
		Total	Expression			Total	Expression		
			Balanced	Imbalanced	%imbalance		Balanced	Imbalanced	%imbalance
Female	LC2/ad	1833572	5071	422	7.68	74265	13	78	85.7
	H1819	1760876	4402	679	13.4	6276	28	39	58.2
	H1975	2190422	5288	591	10.1	4192	0	4	100.0
	H2228	2331330	5904	671	10.2	14467	7	38	84.4
	H2347	2370936	5462	585	9.7	92483	15	140	90.3
Male	A427	1856941	4147	490	10.6	4796	6	1	14.3
	A549	2084037	3422	479	12.3	33667	0	18	100.0
	ABC-1	1423400	2981	381	11.3	4283	3	8	72.7
	H1299	1566125	3346	378	10.2	7107	0	10	100.0
	H1648	2441256	5377	706	11.6	3363	2	21	91.3
	H1650	1136166	2372	343	12.6	2677	1	21	95.5
	H1703	1634715	3645	433	10.6	4480	2	8	80.0
	H2126	1573235	3680	423	10.3	5148	0	6	100.0
	RERF-LC-Ad1	2275585	5607	776	12.2	3557	0	4	100.0
	RERF-LC-Ad2	1975614	4826	541	10.1	4601	2	9	81.8
	RERF-LC-KJ	1892689	4741	546	10.3	3997	0	10	100.0
	VMRC-LCD	1873618	4595	544	10.6	5253	5	8	61.5
Unknown	PC-14	1120101	303	2304	88.4	2177	1	17	94.4
	PC-9	1538099	3825	432	10.2	5973	13	13	50.0
	H322	1222734	3358	433	11.4	3316	2	11	84.6
	II-18	1345778	3039	354	10.4	3733	1	1	50.0
	RERF-LC-MS	1491318	3191	633	16.6	4590	1	4	80.0
	RERF-LC-OK	1855675	4147	513	11.0	74904	31	107	77.5

Supplementary Table S8 PANTHER GO-Slim overrepresentation analysis of regulatory SNVs

Panther GO Slim Biological Process	# reference genes	# matched	# expected	Over/ under	Fold enrichment	P-values
Regulation of gene expression, epigenetic (GO:0040029)	51	5	0.29	+	17.42	2.85e-3
Regulation of nucleobase-containing compound metabolic process (GO:0019219)	534	13	3	+	4.33	2.62e-3
Biosynthetic process (GO:0009058)	1521	23	8.56	+	2.69	2.94e-3
Nitrogen compound metabolic process (GO:0006807)	2018	27	11.35	+	2.38	4.11e-3

Panther GO Slim Molecular Function	# reference genes	# matched	# expected	Over/ under	Fold enrichment	P-values
DNA binding (GO:0003677)	1624	21	9.14	+	2.3	4.88e-2
